# Benefits and risks of orthokeratology treatment: a systematic review and meta-analysis

**DOI:** 10.1007/s10792-024-03175-w

**Published:** 2024-06-21

**Authors:** Lauren Sartor, Damien S. Hunter, Mai Linh Vo, Chameen Samarawickrama

**Affiliations:** 1 Centre for Vision Research, Westmead Institute of Medical Research, Sydney, Australia; 2 Faculty of Medicine and Health, University of Sydney, Sydney, NSW Australia; 3 Department of Ophthalmology, Westmead Hospital, Sydney, NSW Australia

**Keywords:** Orthokeratology, Axial length, Myopia, Microbial keratitis, Contact lens

## Abstract

**Background:**

This meta-analysis reviews the evidence for the risks and benefits associated with orthokeratology (OK) treatment compared with other methods of myopia control in children and adults.

**Methods:**

A systematic search of Cochrane Central Register of Controlled Trials, Pubmed, Embase and Ovid was conducted from database inception to 22nd August 2021. Studies that reported on risks, visual and ocular biometric effects of OK in patients > 5 years of age with myopia (− 0.75 to − 6.00D) were included. Main outcomes are change in axial length and any adverse event.

**Results:**

Fourty-five papers were included in this systematic review and meta-analysis. The quality of data was variable and of moderate certainty, and selection bias likely skewed the results towards a relative benefit for OK. The rate of axial elongation in children was lower for OK treatment compared to other treatment modalities at one year (MD − 0.16 mm, 95% CI − 0.25 to − 0.07). Rate of change in axial length in children rebounded after OK discontinuation compared to participants who continued treatment (MD 0.10 mm, 95% CI 0.06 to 0.14). Adults and children wearing OK were up to 3.79 times more likely to experience an adverse event when compared with conventional contact lenses (OR 3.79, 95% CI 1.24 to ll.), though this evidence base is underdeveloped and requires additional well-designed studies for substantial conclusions to be drawn.

**Conclusions:**

OK arrests myopia progression while in use, however, there remain unanswered questions about the optimal duration of treatment, discontinuation effects and long-term risk for adverse events.

**Supplementary Information:**

The online version contains supplementary material available at 10.1007/s10792-024-03175-w.

## Introduction

Myopia is one of the World Health Organisation’s ten priority eye diseases [1], with global prevalence projected to rise from 34% in 2020 to 50% in 2050 [2]. School aged children in East Asian countries are among the worst affected [3] with prevalence rates reported to be as high as 84% in Taiwanese school children [4]. High myopia is associated with significant risk of complications such as myopic retinal disease, cataract, glaucoma, and retinal detachment [5–8]. Orthokeratology (OK) is a form of overnight rigid gas-permeable (RGP) contact lens which reshapes the curve of the cornea to both correct myopic refractive error in adults and reduce myopic progression in children. The lenses use a reverse geometry design that flattens the central cornea through central epithelial thinning and adjacent mid-peripheral thickening [9–11]. The resulting effect is a temporary reduction of myopic refractive error centrally [9] and the induction of myopic defocus on the peripheral retina [12]. The mechanisms by which OK slows the progression of myopia are still in contention, some of the factors postulated to regulate axial elongation include peripheral defocus [13] increase in corneal higher-order aberrations [14–16] and choroidal thickening [17–19]. Preliminary data for the efficacy of OK has shown favourable improvements in refractive error and axial length measures, though the initial body of evidence has come from case–control and cohort studies with fewer high-quality RCTs contributing data [20]. Systematic review and meta-analyses are valuable in consolidating the evidence from small studies and in determining the effect of OK on myopia progression.

The complications of OK are less well documented and mild side effects such as lens binding and central corneal staining have been described [21, 22]. Microbial Keratitis (MK) is a rare but vision threatening infection of the cornea, and overnight soft contact lens use is recognised to increase the risk of developing MK [23, 24]. Over the past 20 years numerous case reports [25–28] and hospital audits [29, 30] describing MK and acanthamoeba keratitis (AK) in OK wearers have been published, raising concerns about the safety of OK in paediatric wearers. One of the largest studies investigating the relative risk of MK between children and adults is a retrospective post-market surveillance study of 640 adult and 677 paediatric orthokeratology wearers in the USA [31]. The estimated relative risk of MK in adults is 0 per 10,000 patient-years (95% CI: 0–31.7), and in children 13.9 per 10,000 patient years (95% CI: 1.7–50.4). Despite being the largest study published the response rate was 43% of practitioners surveyed and length of follow-up was shorter in adults than children. Additionally, the study was underpowered to detect a rate difference of less than 50 cases in 10,000 patient-years. The rate of MK reported in children is comparable to that seen in estimates for overnight soft contact lenses 19.5–24.5 per 10,000 wearers and greater than estimates of MK in daily wear of 1.2 per 10,000 wearers [24]. There remains a paucity of sufficiently powered, prospective studies investigating the incidence of MK in OK wearers [32–34].

The primary aim of this systematic review and meta-analysis is to evaluate the efficacy of OK as a treatment for myopic progression in children with a secondary aim of assessing the safety profile of OK lenses in both children and in adults. To investigate the efficacy and safety of OK as treatment for myopic progression in children, studies examining the rate of axial elongation and adverse events in OK compared with no-treatment, spectacles, and soft contact lenses (SCL) were included. Studies in adults reporting on change in subjective refraction, corneal power, and adverse events between OK and spectacles, rigid and soft contact lenses or laser-assisted in situ keratomileusis (LASIK) were included. Additionally, studies comparing satisfaction with vision for between the treatments in both children and adults were included.

## Methods

This systematic review and meta-analysis was conducted in line with the Grading of Recommendations, Assessment, Development and Evaluation (GRADE) approach [35].

### Eligibility criteria

All studies comparing OK treatment for ≥ 1 month with non-OK treatment (including single vision or multifocal spectacles, single vision or multifocal contact lenses or LASIK or atropine) or discontinuation of OK treatment for ≥ 1 month, in participants ≥ 5 years of age, with myopia − 0.75 D to − 6.00 D, and astigmatism < − 2.5 D were included. Primary outcomes included change in axial length, the incidence of adverse events, the number of patients who experienced an adverse event. Secondary outcomes are listed in Supplementary Document 1. Randomised and non-randomised studies were eligible for inclusion. Studies were excluded if participants had an underlying ocular disease, if publication was non-English language or if design was a narrative review, systematic review, case-series or case-report.

### Search methods

A search of the Cochrane Central Register of Controlled Trials (CENTRAL), Pubmed, Embase and Ovid with no publication year restrictions was conducted, the final search on 22nd August 2021 (see Supplementary Document 1). In brief, key search terms used were human, orthokeratology, contact lens and myopia.

### Study selection

Identified references for effects of intervention were screened for inclusion via Covidence [36]. Identified references on contact lens prescribing were screened using Excel spreadsheets Screening was independently conducted by two authors. Any disagreements in classification between the review authors were resolved by discussion and consensus, or by input from the senior author.

### Data collection and risk of bias

Two authors independently extracted data for each included study on study design, funding sources and declarations of interest, participant characteristics, intervention and comparator characteristics and quantitative outcomes. Discrepancies in data extraction and calculation of derived data were resolved by consensus. Collated data was exported into Review Manager 5 [37] software by one author, and independently verified by a second author.

Two authors independently assessed the risk of bias of the included studies. Randomised controlled trials were assessed using the *Cochrane Risk of Bias 2.0* tool [38] and non-randomised studies were assessed using the *Risk of Bias in Non-randomised studies of Interventions (ROBINS-I)* tool [39]. All non-randomised studies with a critical risk of bias were excluded from analyses. Two authors independently rated the quality of evidence for each outcome using the GRADE system [35]. For selected outcomes with sufficient studies to include in meta-analyses, relative funnel plots of asymmetry were examined to assess risk of publication bias (i.e. selective reporting of outcomes).

### Data synthesis and analysis

For interventional studies, comparisons were made between both OK and pooled non-OK comparator treatment, and between OK and each individual comparator treatment where there were an adequate number of studies. Three separate comparisons were conducted to examine effects of discontinuation: (1) Parallel OK and discontinuation groups, (2) During OK and after discontinuation (cross-over design), (3) Discontinuation of OK to parallel non-orthokeratology comparator groups.

An adverse event was defined as any report of an undesired treatment effect. Due to inconsistency in reporting of events between studies, the derived data is of the number of participants who experienced an adverse event in each group. For individual complications, for example, corneal staining, meta-analyses were performed if there were ≥ 2 studies reporting on the event in a consistent manner.

Meta-analyses were conducted where there were ≥ 2 studies reporting on the same outcome. Fixed effects models were used if < 3 trials were included in analysis, or for outcomes with heterogeneity *I*^2^ ≤ 20%, and a random-effects model for all others. Sensitivity analyses were conducted for the primary outcome to assess the effect of excluding highly biased studies where numbers permitted. Effects of the intervention were expressed as a mean difference (MD) with 95% confidence intervals (CI) or an odds ratio (OR) with 95% CI, or as appropriate.

## Results

**Fig. 1:**
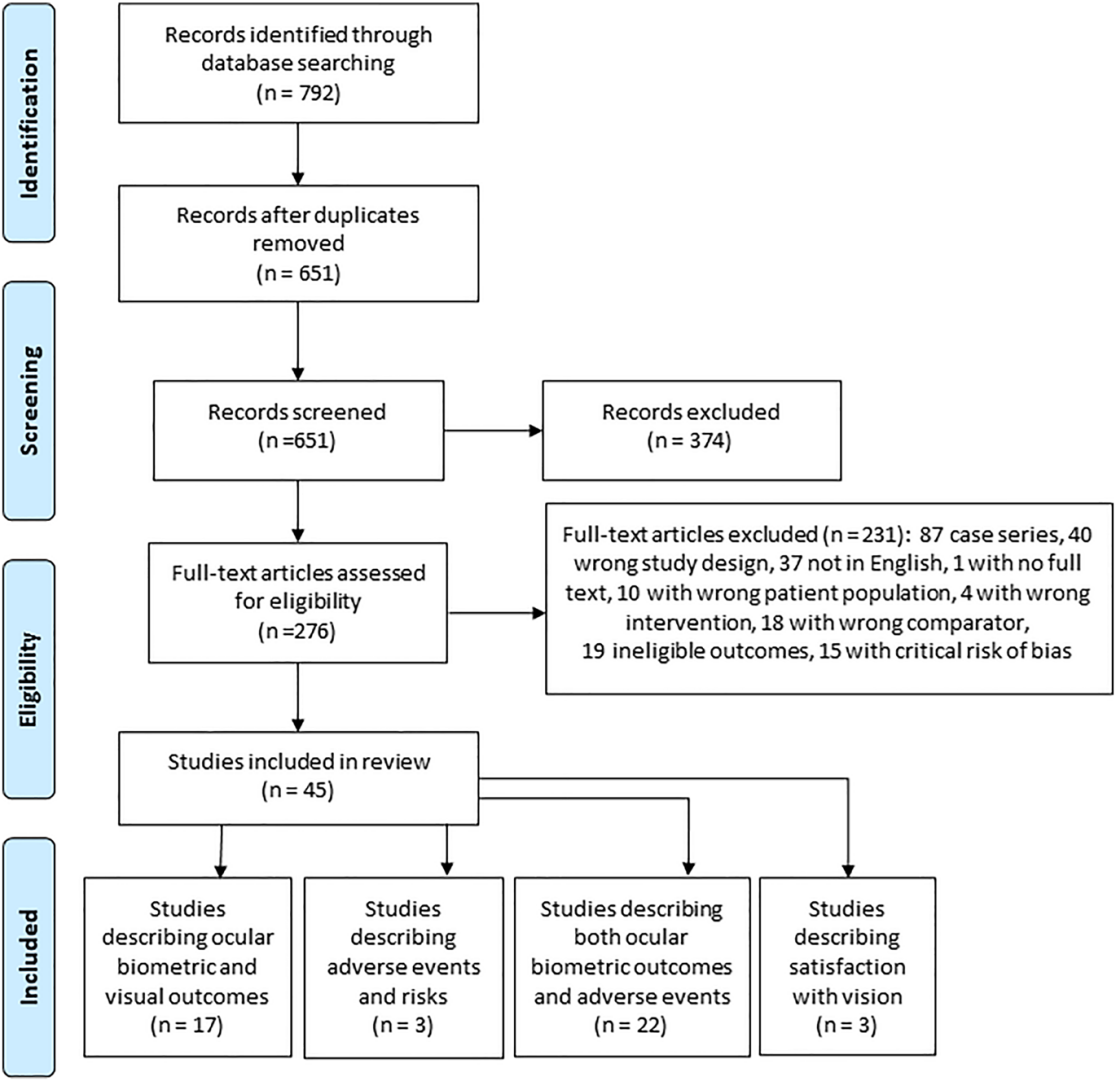
PRISMA diagram of search methods, screening and included studies

Figure 1 illustrates the study selection process. A total of 651 studies were identified after removal of duplicates, of which 45 (17 RCTs and 28 non-randomised studies) were included in this review. Full details of each study are described in supplementary Tables 1 and 2.

### Risk of bias

**Fig. 2:**
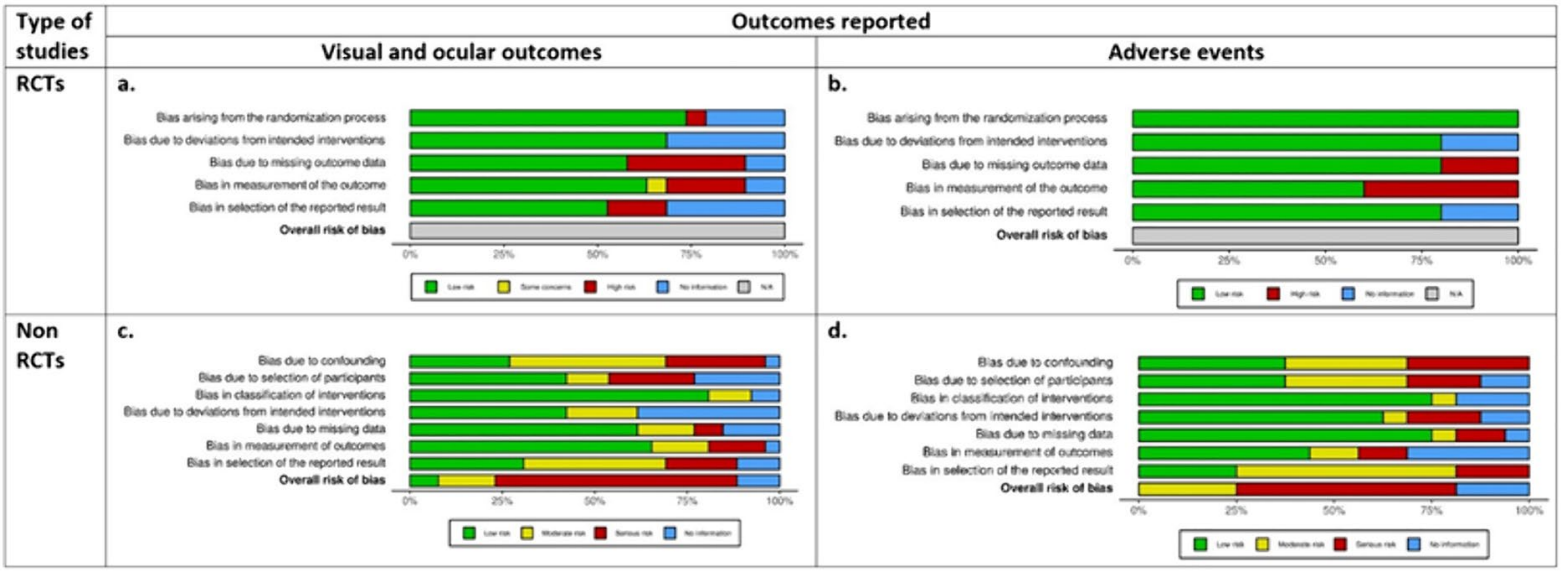
Risk of bias of RCTs and non-RCTs reporting on visual/ocular outcomes and adverse events. Randomized studies’ risk of bias was assessed using the Cochrane Risk of Bias tool; non-randomized studies were assessed by Cochrane Risk of Bias for Non-Randomized Studies tool (ROBINS-I)

Risk of bias for RCTs is summarized in Fig. 2a and b, with detailed results reported in Supplementary Document 2. Of the 17 full length papers which represented 10 studies, bias was assessed for each individual domain. Biometric outcomes were assessed in 17 of the papers and adverse events were assessed in 5 of the RCTs included. Randomization was inadequately described in 4 papers and 1 had a high risk of bias. Missing outcome data domain was judged to be at high risk of bias in 6 papers and unclear in 2 papers, owing to the exclusion of treatment non-responders and dropouts. Measurement of outcome data was judged to be at high risk of bias in 4 studies, of some concern in 1 study, and unclear in 2 papers owing to the masking of participants and outcome assessors, which was unavoidable given the nature of the treatment groups, i.e. OK lens vs. spectacles.

Risk of bias for the 28 non-randomised interventional studies are summarized in Fig. 2c and d. Full ROBINS-I results are reported in Supplementary Document 2. Amongst studies reported on visual and ocular biometrics, only 2 included studies had low risk of bias and 4 had moderate ratings. 17 had serious risk of bias, and 3 provided insufficient information on one or more domains to accurately classify overall risk of bias. No included studies reporting on adverse events had low risk of bias, 4 included studies had moderate ratings, 9 had serious risk of bias, and 3 provided insufficient information on one or more domains to accurately classify overall risk of bias.

Eight studies received funding or support from industry which increased the likelihood of studies reporting the positive effect of change in axial length and increasing the risk of publication bias [40–46]. In some studies the nature of the funding was not fully described [40–42] and in other cases the support was exclusively in the form of contact lenses, solutions and spectacles [44–46], only one study reported no involvement of industry “in the design or conduct of the research” [46]. Some studies had excluded poorly responding participants, suggesting amplification of treatment benefits [41, 43, 47–51]. For example, participants were excluded for under response to treatment [41, 48], three studies excluded participants who did not complete the intended length of lens wear without detailing any number or reasons for exclusions [50–52]. For quality-of-life studies, patients were excluded if they did not demonstrate satisfactory improvements in vision for all interventions [47].

Rare adverse events were unlikely to be detected due to short study durations and small sample sizes. Methods by which adverse events were classified typically were not reported, and use of standardised measurement tools was uncommon (e.g. Dry Eye Questionnaire). Adverse events were typically reported in an ad hoc fashion, even by RCTs.

### Outcome measures

**Fig. 3:**
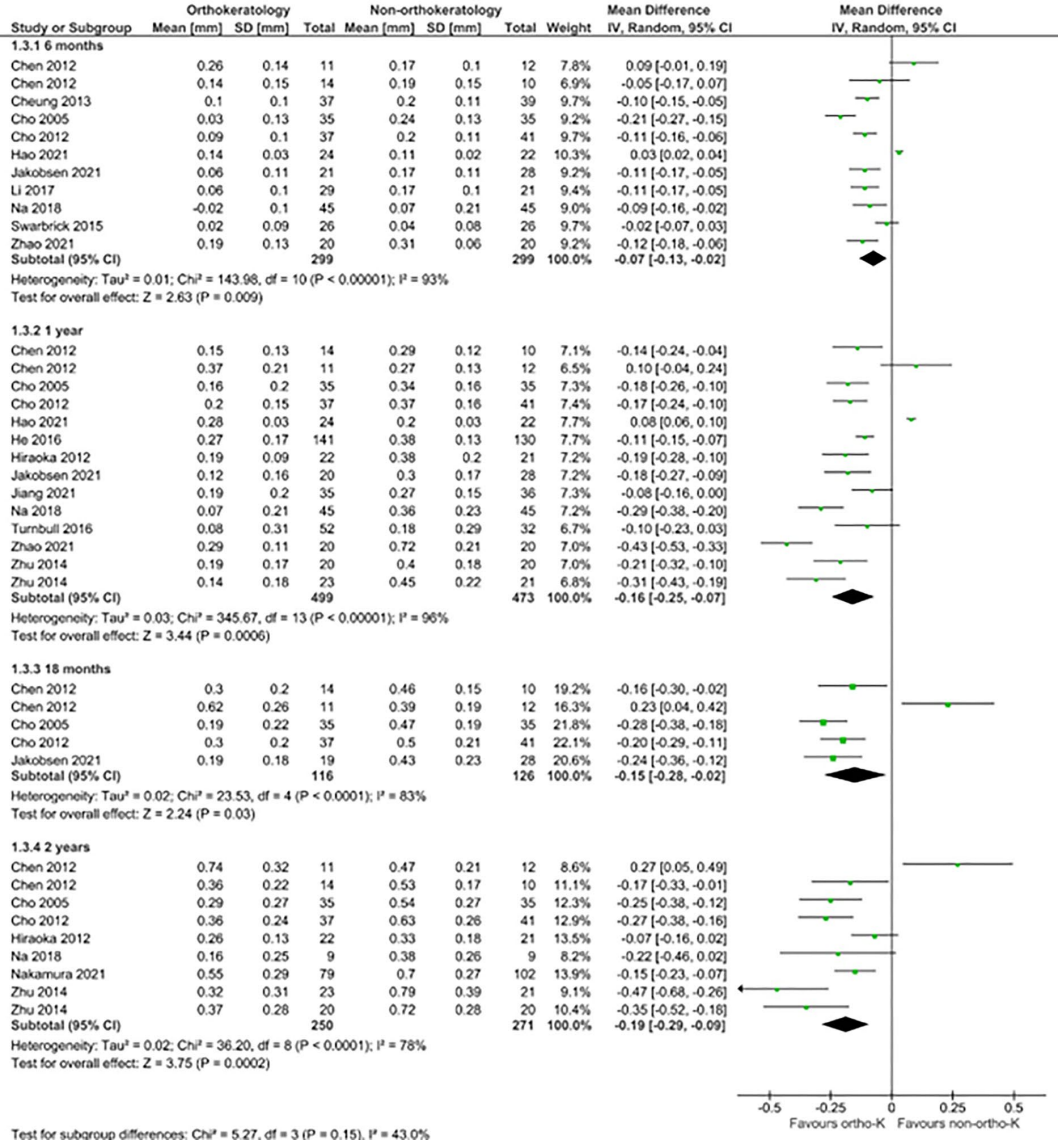
Forest plot comparing change in axial length in orthokeratology vs comparator treatments at 6, 12, 18 and 24 months. Outcomes were reported solely in studies with children as participants. Two studies (Chen 2012 [54] and Zhu 2014 [50]) included multiple orthokeratology and comparator subgroups, data for which were separately extracted and included in analysis

**Fig. 4:**
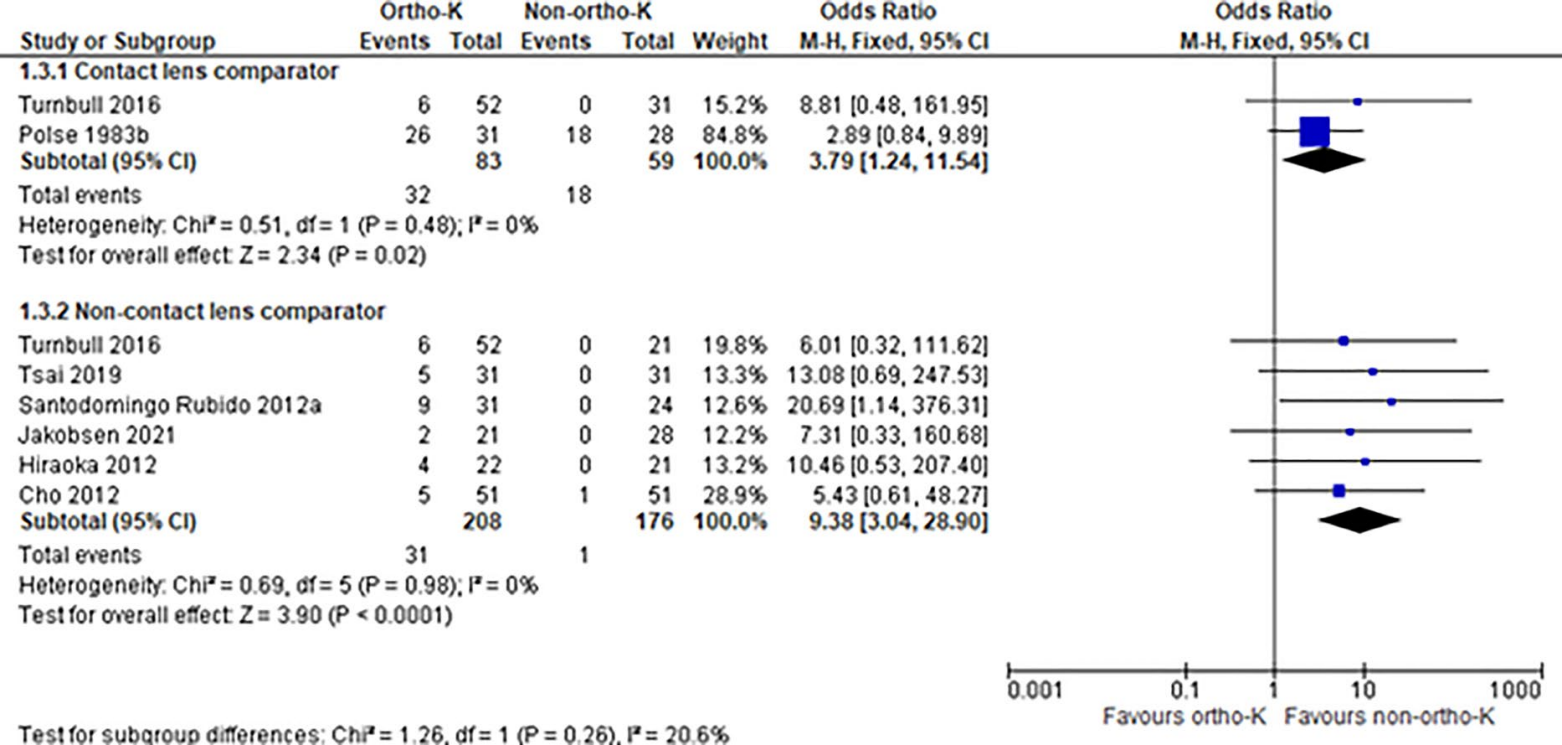
Forest plot comparing number of participants experiencing any adverse events in orthokeratology compared with non- orthokeratology treatments. Contact lens groups consisted of adult and paediatric participants and non-contact lens groups consisted of paediatric participants only

The main effects of intervention (OK vs non-OK) on the primary outcome measure of axial length and adverse events are illustrated in Table 1 and Figs. 3 and 4. The certainty of evidence as rated using the GRADE is presented in Table 2. Primary and secondary outcomes are reported in Supplementary Tables 4–18, including subgroup analyses for effects of time point, participant age and comparator treatment modality where sufficient studies were available. Results of analyses for primary outcomes are as follows.

**Table 1:** Summary of findings for change in axial length compared with all non-OK groups at four time-points in a paediatric population. The adverse events are presented as an OR between the OK group and contact lens comparator groups, which contain both adult and paediatric populations. The discontinuation of OK is compared with continuation of OK and a no treatment control group which was measured in paediatric patients

Comparison groups	Outcome measure	No. of participants (Studies)	Follow up time	Effect estimate (95% CI)
OK vs non-OK treatment	Change in axial length (mm)	598 (10)	6 months	MD 0.07 mm Lower (95% CI 0.02 mm lower to 0.13 mm lower)
		972 (12)	1 year	MD 0.16 mm Lower (95% CI 0.07 mm lower to 0.25 mm lower)
		242 (4)	18 months	MD 0.15 mm Lower (95% CI 0.02 mm lower to 0.28 mm lower)
		521 (7)	2 years	MD 0.19 mm Lower (95% CI 0.09 mm lower to 0.29 mm lower)
OK vs contact lenses	Adverse events (n participants)	142 (2)	Completion of study	OR 3.79 (95% CI 1.24 to 11.54)
Discontinuation vs OK (parallel group)	Change in axial length (mm)	79 (2)	6–7 months	MD 0.10 mm higher (95% CI 0.06 mm higher to 0.14 mm higher)
Discontinuation vs non-OK	Change in axial length (mm)	78 (2)	6–7 months	MD 0.06 mm Higher (95% CI 0.02 mm higher to 0.10 mm higher)

**Table 2:** Grading of recommendations, assessment, and evaluation (GRADE) rating for primary outcomes

Outcome and comparison groups	Risk of bias	Imprecision	Inconsistency	Indirectness	Publication bias	Quality of evidence
Change in axial length (mm)						
*OK vs Non-OK treatment RCT*	Unclear^a^	Low	None^e^	Low	Some risk^i^	⊕ ⊕ ⊕ ◯ Moderate
*Observational*	High^b^	Low	None^e^	Low	Some risk^i^	⊕ ⊕ ◯◯ Low
*Discontinuation vs OK or Non-OK*						
*RCT*	Unclear^c^	Low	None^f^	Differences in intervention^g^	Some risk^i^	⊕ ⊕ ⊕ ◯ Moderate
Adverse events						
*OK vs Non-OK*						
*RCT*	Unclear ^a^	Some risk^d^	None	Differences in outcomes^h^	Some risk^i^	⊕ ⊕ ◯◯ Low
*Observational*	High^b^	Some risk^d^	None	Differences in outcomes^h^	Some risk^i^	⊕ ⊕ ◯◯ Low

High: Further research is very unlikely to change our confidence in the estimate of effect

Moderate: Further research is likely to have an important impact on our confidence in the estimate of effect and may change the estimate

Low: Further research is very likely to have an important impact on our confidence in the estimate of effect and is likely to change the estimate

Very low: We are very uncertain about the estimate

^a^Risk of bias in RCT high—unclear information regarding selection, randomization, masking, selective reporting of baseline data

^b^Risk of bias in Observational study high–confounding, selection of participants, dropouts

^c^Risk of bias in RCT–masking of participants, one study did not account for dropouts

^d^Wide confidence intervals, small sample size

^e^I^2^ values > 80% when examining RCTs only and observational studies only at 1 and 2 years, though sample sizes noted to be small

^g^Differences in the OK comparison group, Swarbrick 2015 uses group commencing OK whereas Cho 2017 uses group who are continuing OK wear

^h^Variation in RCTs and Observational studies regarding the adverse events that were measured and some reported as incidental findings

^i^Funnel plots show relative asymmetry, many studies were conducted in collaboration with or sponsored by organisations that produce contact lenses

### Change in axial length

#### Non-OK comparators

Sixteen studies compared change in axial length between OK and non-OK comparator treatments at 10 different time points, solely in paediatric participants. Figure 3 reports effects of OK vs non-OK treatments after 6, 12, 18 and 24 months of treatment. High quality data was available for 6-month and 12-month analyses and did not show differences in axial length progression at 6 months but did show benefit at 12-months (see supplementary Table 5; 6 month MD −0.11 mm 95% CI −0.22 mm to 0.00 mm and 12-month MD −0.18 mm 95% CI −0.24 mm to −0.12 mm). For extended follow-up, only low-quality data was available for analysis and differences in axial length were seen at 18-months (MD −0.15 mm, 95% CI −0.28 mm to 0.02 mm) and 24-months (MD −0.19 mm 95% CI −0.29 mm to −0.09 mm).

#### Compared with discontinuation

Two studies (81 eyes) compared the changes in axial length between orthokeratology use and discontinuation of treatment after 3, 6 or 7 months [46, 53] in participants aged between of 9–14 [53] and 10.8–17 years [46]. At 6–7 months after discontinuation, there was a significant increase in the axial length of the discontinuation group when compared with continuation of orthokeratology treatment (MD 0.10 mm; 95% CI: 0.06 to 0.14 mm). When compared with non-OK controls, the axial length in the discontinuation group was also greater for the same timepoint (MD 0.06 mm, 95% CI 0.02 mm to 0.10 mm) at 6–7 months.

### Adverse events

Figure 4 summarises the number of participants in each study who experienced an adverse event. A greater number of OK wearers experienced an adverse event than contact lens controls (OR 3.79, 95% CI 1.24 to 11.54 participants) or non-contact lens controls (OR 9.38, 95% CI 3.04 to 28.90 participants). Adverse events were not consistently measured or reported amongst the studies though subgroup analyses were conducted where possible (full results in Supplementary Tables 11–13). The events measured included corneal erosion [52, 55], corneal staining [41, 50, 56–59], dimple veiling [57], dry eye [60], epithelial iron deposition [61], lens binding [46, 62] and corneal infiltrates [52]. MK was not reported in any interventional studies. The funnel plot (Supplementary Fig. 2) shows relative symmetry. Analysis of RCTs only reveal an OR 3.81 (95% CI 1.40 to 10.34), with two studies representing children and with spectacle comparators and one representing adults with a CL comparator group.

The contact lens comparator group in Turnbull et al. consisted of children with a follow up period of one year [62]. Over this time there was one case of a central corneal epithelial defect and five cases lens adherence and pain with removal. The Berkely study published by Polse et al. [56] reported the percentage of adult participants with corneal oedema, central and limbal staining and the corresponding grade for each (1–3). It is reported as a pooled percentage of each grade, taken from all visits, separately for morning (AM) and afternoon (PM) examinations. A majority of participants have either grade 0 or 1 of each complication, with grade 2 oedema seen in 4.6% of the OK group and 3.8% of the CL control group, and grade 3 oedema seen in 0.2% of the control group. Grade 3 central staining was seen in 0.2% of the OK group and none in the control group. The total number of follow-up visits for review of complications was also reported, of which there were 76 for the ortho-K group (26 of 31 participants), and 56 for the comparator group (18 of 28 participants).

The studies reporting non-contact lens comparison groups were all conducted with children. Turnbull et al. [62] compared OK with spectacles, Tsai et al. [63] compared OK in one eye with a fellow emmetropic eye where the adverse events included three cases of mild superficial punctate keratopathy (SPK) and two mild allergic reactions, but the inciting cause was not described. Santodomingo-Rubido et al. [57] examined OK compared with spectacles over two years. In the treatment group they describe 5 cases of corneal erosions, 2 of corneal staining, 2 of papillary conjunctivitis, 1 case of peripheral ulcer and 1 case of dimple veiling in addition to blepharitis, bacterial conjunctivitis and hordeolum. Jakobsen et al. [64] is an RCT which examined OK vs spectacles over 18 months and describe 2 participants with corneal staining of grade 3 severity, and no adverse events in the spectacle group. Hirakoa et al. [55] followed children with OK compared with spectacles over 5 years and reported moderate SPK in three patients and one mild corneal erosion in the OK group. Cho et al. [41] compared OK with spectacles over two years and noted one case of corneal inflammation in the control group, in the OK group there were three cases of persistent and significant corneal staining, one case of increased conjunctival hyperemia and one case of chalazion.

## Discussion

This systematic review and meta-analysis has shown that OK is moderately effective at slowing myopia progression in children after one year of use, in concordance with other meta-analyses [65–67]. However, this is tempered by preliminary evidence of a rebound in axial length growth following cessation of OK. Adverse events are more common in OK wearers than for CL or spectacle wearers, though the events that have been reported are relatively benign and reversible.

Slowing the progression of myopia in children may help to prevent progression to high myopia [68] and its associated complications in later life, including myopic macular degeneration and retinal detachment [69, 70]. The results demonstrate that OK is an effective method of slowing axial elongation, with an effect of -0.19 mm over two years of wear, which is more conservative than that seen in other reviews [65–67]. Though beyond the scope of this review, there is emerging research investigating the effects of combined OK and atropine therapy [71, 72], which may be of greater benefit to children with small pupils. The review of safety data has highlighted that adverse events have not been as rigorously examined as the biometric effects for OK. Many of the included studies did not assess safety as a primary outcome and the risk of bias in non-randomised studies was high for the measurement of adverse events (Supplementary document 2). When comparing OK to spectacle or no-treatment, the OR for an adverse event is comparable to that reported in other meta-analysis OR 8.87 (95% CI 3.79–20.74) [66]. When compared with CL comparator groups the OR is more conservative (3.79, 95% CI 1.24 to 11.54), though only two studies were suitable for the calculation. The adverse events that were reported recovered with a brief discontinuation of lens wear in all but one study, where OK lens wear was discontinued for three cases of significant corneal staining and one of conjunctival hyperaemia [41]. Of the complications represented in the forest plot, 8 cases of corneal erosions or ulcer were described, which were the more serious adverse events reported. Erosions have been postulated to increase the risk of corneal infection in CL wearers [73], though there were no reports of MK in the studies reviewed. Most of the reviewed studies had samples of < 50 patients per group and lacked the power to detect rare events such as microbial keratitis, which has been noted by other authors [20]. CL wear is a recognised risk factor for microbial keratitis [74], which is exacerbated by overnight use [24, 75]. In adequately powered studies we should expect to see a small number of MK cases to this baseline level. Due to the inconsistent measurement of adverse outcomes and relatively short duration of follow-up, and unexplained drop-outs or exclusions, conclusions regarding the safety of OK cannot be confidently drawn from the data available.

MK, and especially AK, are rare but serious complication that can have poor outcomes even if diagnosed and managed early [76]. Reports of MK in OK wearers continue to be published [27], and highlight its deleterious impacts on children and adolescents including long courses of antimicrobial therapy, hospitalisation, months spent out of school impacting education, and the permanent vision loss and visual disturbance resulting from corneal scarring [27, 28]. One study has attempted to estimate the risk of MK in Russian children wearing OK lenses, [77] by taking the cases of MK at Moscow children’s hospital and comparing it with an extrapolated number of lens fits from a network clinics that specialise in OK lens prescriptions. The incidence rate was estimated to be 5.3 cases per 10 000 patient years, based on a discontinuation rate of 10%. A post-market surveillance study carried out in the United States of America, [31] estimated the incidence of MK in children to be 25.2 cases per 10,000 patient years based on 12-months of wear, though, even in this study the authors acknowledge the sample was small and poorly powered. Additional risks for AK include poor hygiene practices including storing CL in tap water or using it to top off solutions in the case [24, 78], practices which are strongly cautioned against when initiating OK therapy [33]. Given the best estimates of incidence can only be extrapolated from secondary data, well-designed studies of adequate power and duration or utilisation of multi-centre clinical registries are required to draw conclusions regarding safety.

Low-dose atropine is an alternative treatment for myopic progression which is widely prescribed among children [79]. It has been shown to retard axial growth to a degree comparable to OK, with a dose–response effect [80, 81]. The adverse event profile for atropine is different to OK, with the most common adverse event reported being photophobia, followed by difficulty reading, eye pain and headaches [81]. Similar to the OK literature, the studies examining adverse events are few and of short duration, though the risks for corneal infection that are inherent in CL wear do not apply to this modality. To our knowledge there are no studies that directly compare the outcomes of atropine monotherapy with OK monotherapy. Given the effectiveness of atropine drops and the relatively milder side effect profile, we suggest that atropine be considered the first line therapy with OK adopting a secondary role.

The discontinuation of OK treatment did reveal an increase in axial elongation when compared with continuation of treatment and control groups, though this difference was small and of uncertain clinical significance. The two studies summarised in this analysis contain a small number of participants and were of short duration; it remains unclear for how long the trend in greater axial length growth will continue before stabilising. In the case of one study baseline differences existed between the treatment and control groups, where the discontinuation group had a higher axial length than the continuation and control groups (24.94 ± 0.89; 24.72 ± 0.90; 24.69 ± 0.88, respectively) [53]. Weather the relatively rapid axial elongation observed is an underlying difference in the speed of progression between the groups cannot be determined from this data alone, though the finding that the change in axial length reduced upon resumption of OK does suggest that the effect is moderated by OK lens wear.

As myopic progression stabilises in mid adolescence [82], OK offers the primary benefit of refractive correction for adults. There were an insufficient number of studies for a pooled analysis of improvements in refractive error, though individual studies have shown similar improvements in visual acuity and in vision related quality of life between OK and SCL [45], an improvement in sphere equivalent when compared with RGP lenses [83], and that both OK and LASIK offer improvements in uncorrected visual acuity [84]. A minority of studies included in this review measured quality of life scores. When compared with SCL, adult satisfaction scores were lower in OK (Supplementary Table 10) and overall satisfaction was no different between OK and LASIK groups [47, 85]. For adults who find daywear CLs and spectacles unappealing, OK is a reversible option for correction, and its success is dependent upon compliance with treatment. LASIK, or other refractive surgical procedures, are a long-term alterative for refractive error correction that is well tolerated [86–89], but has its own risk profile of intraoperative and post-operative complications, with microbial keratitis estimated to occur in 0.005%-0.034% of cases [90].

Considering the differing goals of treatment and the volume of evidence in children compared with adults, the risk–benefit profile of OK is varied between these groups. Slowing myopic progression in children may reduce the risk of complications in later life [69, 70]. It may also reduce the lifetime cost of vision correction and of managing complications to both the patient and the community [91]. This needs to be balanced with the risk for serious, vision threatening infection which is inherent in overnight CL wear and with a therapy that requires prolonged use over several years to be effective. The evidence for alternatives such as atropine is preliminary, but if tolerated is also effective in reducing axial length and without the same risk for infection. In adults, where the purpose of OK is to manage refractive error, the ratio of risk to benefit may be shifted, as overnight CL wear comes at a greater risk for infection than daily wear [24] and similar to that of LASIK, [92] which is a permanent alternative.

## Limitations

Owing to the small number of RCTs (10 RCT vs 34 cohort and case–control) prospective and retrospective case–control studies with diverse study design were included. Heterogeneity was high in the primary outcome change in axial length I^2^ > 75%. The differences in study design precluded subgroup analysis for many of the efficacy and adverse event outcomes.

A risk of bias assessment was performed to exclude studies with a critical risk, though bias was not totally unavoidable, and there remained several studies reporting on efficacy that excluded poorly responding patients, [41, 48] that demonstrated a selection bias [50–52] and most were not adequately designed to detect rare adverse events. Much of contact lens research is supported by industry, which can be through the donation of products or funding, and a small number did not fully disclose the nature of their funding.

Since some of the earlier OK studies were published contact lens fitting technology has changed and hygiene practices have improved [33]. The prevalence of adverse events may be much lower than earlier reports, however, these trends in practice are challenging to quantify.

## Conclusion

The research surrounding OK is primarily focused on its efficacy as a treatment for myopic progression in children, for which it can reduce axial elongation by approximately 0.19 mm over two years. This review has highlighted several important areas for further research including the effect of discontinuation on axial elongation, which will have implications for the optimum duration of treatment, and the potential benefits of synergistic therapy. Well-powered studies of safety are required to improve the rigour of the evidence base which is currently underpinning OK therapy and needed before valid conclusions can be drawn regarding its safety. Despite the lag in safety research, OK is growing in popularity amongst practitioners for use in children [93, 94]. Education in both children and adults who are starting OK therapy should come with strict guidance on hygiene practice and on the importance of compliance. OK is a promising therapy for the management of myopia and although the evidence base guiding its use is limited, it can be strengthened with larger, well-designed trials addressing these areas.

## Supplementary Information

Below is the link to the electronic supplementary material.

Supplementary file1 (DOCX 1997 KB)

Supplementary file2 (DOCX 286 KB)
