# Supplementary material for: Benefits and risks of orthokeratology treatment: a systematic review and meta-analysis
Source: Int Ophthalmol. 2024 Jun 21;44(1):239. doi: 10.1007/s10792-024-03175-w (PMC11192849; doi:10.1007/s10792-024-03175-w)
Supplement: Supplementary file 1 — Supplementary file1 (DOCX 1997 KB) [file 10792_2024_3175_MOESM1_ESM.docx]

International Ophthalmology

s

**Benefits and risks of orthokeratology treatment – a systematic review and meta-analysis**

Lauren Sartor* MChD,^2,3^ Damien S. Hunter* PhD,^1,2^ Mai Linh Vo* MD,^2^ Chameen Samarawickrama FRANZCO ^1,2,3^

^1^Centre for Vision Research, Westmead Institute of Medical Research, Sydney, Australia

^2^Faculty of Medicine and Health, University of Sydney, New South Wales, Australia

^3^Department of Ophthalmology, Westmead Hospital, New South Wales, Australia

**Corresponding Author:**

Associate Professor Chameen Samarawickrama

Email: chameen.sams@sydney.edu.au

**Contents**

Supplementary Document 1 - Detailed Methods 3

Supplementary Document 2: Risk of bias in randomized and non-randomized studies 7

Supplementary Document 3: Adverse Events 17

References 22

**Supplementary Document 1. Detailed Methods**

This systematic review and meta-analysis was conducted in line with the Grading of Recommendations, Assessment, Development and Evaluation (GRADE) approach [1].

***Eligibility criteria***

*Effects of intervention*

Inclusion criteria

- Participants ≥ 5 years of age, with myopia −0.75 D to −6.00 D, and astigmatism < 2.5 D).
- Interventions: OK monotherapy treatment for ≥ 1 month
- Comparator treatment: non-OK treatment including single vision or multifocal spectacles, single vision or multifocal contact lenses or LASIK or atropine or discontinuation of OK treatment for ≥ 1 month
- Outcomes: quantitative data reported for
  - Primary outcomes: Change in axial length, Incidence of any adverse event, Participants experiencing adverse events, Microbial Keratitis
  - Secondary outcomes: refractive sphere, spherical equivalent refractive error, refractive cylinder, uncorrected visual acuity, best-corrected visual acuity, change in corneal curvature, corneal thickness, patient satisfaction, corneal staining, dimple veiling, dry eye, epithelial iron deposit, intraocular pressure (IOP), lens binding

Exclusion criteria

- Underlying ocular disease (e.g. retinopathy, prematurity, genetic disease)
- No English language full-text available
- Study design: narrative review, systematic review, case-series, case-report

***Electronic searches***

A search of the Cochrane Central Register of Controlled Trials (CENTRAL), Pubmed and Embase Ovid, with no publication year restrictions was conducted, the final search on August 22^nd^ 2021.

To identify studies for effects of intervention, search terms were as follows, with adjustment as appropriate for each database (eg. Emtree terms for Embase): (Human AND (Paediatric or pediatric or child*) AND (adult or geriatric)) AND ((Orthokeratology or OK or Orthok or Corneal Refractive Therapy) AND (Contact lens* OR (MeSH descriptor: (Contact Lenses) explode all trees OR (Atropine)) AND ((MeSH descriptor: (myopia) explode all trees OR (myop* or short sight*) OR (astigmat* or refractive error) OR (MeSH descriptor: (Astigmatism) explode all trees) OR (MeSH descriptor: (Visual Acuity) explode all trees) OR (accommodat* or acuity) OR (progress* or slow* or retard* or funct*) OR (discontinuat* or rebound*) OR (adverse* or decentrat* or infect* or corneal ring*)) AND (Limits: Trials)

***Study selection***

Identified references for effects of intervention were screened for inclusion via Covidence [2]. Identified references on contact lens prescriptions were screened on a custom-built Excel tool. Data extraction for each type of data occurred in the programs. Screening occurred independently by two authors. Any disagreements in classification between the review authors were resolved by discussion and consensus, or by input from a third author.

***Data collection***

*Effects of intervention*

Data was extracted according to the following categories:

- Study design and type as per as per [3], country, setting, year of publication, funding sources and declarations of interest
- Participants characteristics (age, number)
- Details of intervention and comparators
- Effects of treatment for outcomes of interest

When required, original investigators were contacted to resolve ambiguous data or methodological questions.

Due to inconsistent reporting of overall incidence and severity of adverse events, this was manually calculated by authors, with adverse event defined as all reports of undesired treatment effects, regardless of severity. Derived data took the form of either overall incidence of adverse events in each study or number of participants experiencing adverse events, depending on contributing data.

Data on infection was handled separately in hospital audits, which reported incidence of patients presenting with either MK or AK, rather than incidence of each in the treatment and comparator groups.

Any discrepancies in data extraction and calculation of derived data were resolved by consensus. Collated data was exported into Review Manager 5 [4] software by one review author, and verified by a second author.

***Risk of bias assessment for included studies***

Two review authors independently assessed the risk of bias of the included trials. Randomised controlled trials were assessed using the Cochrane Risk of Bias 2.0 tool [5] and non-randomised studies were assessed using the Risk of Bias in Non-randomised studies of Interventions (ROBINS-I) tool [6]. All non-randomised studies with a critical risk of bias were excluded from analyses. Two authors independently rated the quality of evidence for each outcome using the Grading of Recommendations, Assessment, Development and Evaluation (GRADE) system [1]. This included evaluations for each outcome on study limitations, inconsistency, indirectness, imprecision and publication bias. The GRADE profiler software was used to document the evaluations and to create a summary of findings table (available at: www.gradeworkinggroup.org).

For selected outcomes with sufficient studies to include in meta-analyses, relative funnel plots of asymmetry were examined to assess risk of publication bias (i.e. selective reporting of outcomes).

***Data synthesis and analysis***

The preferred unit of analysis was the eye and individual or treatment group were used where appropriate.

*Interventional studies*

Comparisons were made between OK and non-OK comparator treatments at 6 months, 12 months, 18 months and 24 months, with subgroup analysis to compare to individual comparator treatments where there was sufficient data. Three separate comparisons were conducted to examine effects of discontinuation where data permitted:

- Parallel OK and discontinuation groups.
- During OK and after discontinuation (cross-over design)
- Discontinuation of OK to parallel non-orthokeratology comparator groups.

Meta-analyses were conducted when possible. Fixed effects models were used if <3 trials were included in analysis, or for outcomes with heterogeneity I^2^ ≤20%, and a random-effects model for all others. Where a meta-analysis was inappropriate due to inadequate contributing studies individual results were not reported.

Where there were sufficient studies, sensitivity analysis were conducted for primary outcome to assess effects of excluding highly biased studies (RCTs which systematically excluding non-responders from reported data and non-RCTs with an overall serious risk of bias).

**Supplementary Document 2: Risk of bias in randomized and non-randomized studies**

**Risk of bias in randomized studies**

Risk of bias for RCTs is summarized in Figure 2a and 2b, with detailed results reported in Figure 1. Amongst the n = 17 full length papers representing 10 studies, there were different risks of bias in reporting of visual outcomes and adverse events, which were assessed separately. Each individual domain of bias for included papers is assessed as followed.

While bias for the randomization process was typically low (n = 12 low in bias, n = 1 high risk, n = 4 no information) and allocation concealment was frequently inadequately described. One study [7] was judged as having a high risk of bias as the authors did not describe the randomization process, but stated that some patients refused to be randomized and were permitted to choose their groups. Cho 2017 was noted to be at risk of selection bias to inherent differences between treatment groups. This study recruited participants who had participated in previous trials by the researchers and as a result there were likely to be differences between groups. This may explain the differences between treatment groups which were present prior to the commencement of the study and also affected the results of treatment discontinuation [7].

Blinding of participants and personnel was variable but we judged more to be at a high risk of bias for the majority of papers. This was unavoidable due to the nature of some of the comparator groups used (i.e. contact lenses vs spectacles), and the differences in timing of wear between different contact lenses making. The Berkeley Study [8] was the only study which was successfully blinded participants due to OK lenses being worn by participants during the day. The dispensing schedules of RGP comparator group lenses was also designed to mimic the dispensing schedule of OK lenses. Blinding of outcome assessment was more varied, in four studies the high risk of bias was due to the outcomes assessed being self-reported quality of life surveys and self-reported adverse events incidence [9-12], while in the fourth, the authors reported, “The unmasked examiners knew if a subject was on OK treatment from the good unaided vision, the low (residual) refractive error, the typical topographic maps, and ocular signs (i.e., pigmented arc) observed in slit-lamp biomicroscopy. However, OK did not present any particular identifying features during axial length measurement and the examiner performing the measurement could be masked”[13]. Low risk of bias in measurement of the outcome did not necessarily entail full blinding of outcome assessors, but rather that blinding of outcome assessors was unlikely to have an effect on data collection, e.g. measurement of axial length with automated machines.

Five studies were judged to be at high risk of bias for reporting incomplete outcome data (n = 11 low risk of bias, n = 6 high risk of bias, n = 2 no information). The full-texts reporting on the ROMIO study [13, 14] were judged to have a high risk of bias on this outcome as they excluded non-responders to treatment: “Subjects who failed to achieve satisfactory results, including decentered treatment zone and/or poor ocular response despite repeated (three times) lens modifications, and non-compliance to the wearing schedule, were excluded”[13]. These papers did not report any of the data collected on these participants including baseline measures, and there may be differences in these and in adverse events between the group of participants that completed the study and those who were excluded. Cho 2017 [7] similarly did not account for the difference between participants completing and dropping out of the study, which was possible at baseline given the participants for this trial were continuing following previous trials ran by the group. Lipson and colleagues [9, 10] were judged to have a high risk of bias due to high attrition rate, which in a quality of life study we deemed likely to bias results as participants with adverse outcomes or low quality of life were unlikely to continue treatment and remain in the study.

Three papers had a high risk of bias for selective reporting (n = 10 low risk of bias, n = 3 high risk of bias, n = 6 no information). We judged the papers reporting outcomes for the ROMIO trial[13, 14] to have a high risk of bias for excluding participants that did not achieve satisfactory results to OK treatment, with one paper entirely failing to mention the exclusion of participants and fully describe data collected during the study [14]. We judged Ritchey 2005 [11] to have high risk of bias for selective reporting, as data was only reported at the 3 month time point, however, “subjects were followed up for 3 months and were examined at 1-day, 1-week, 1-month, and 3-month intervals after a successful lens fit was achieved” [11].


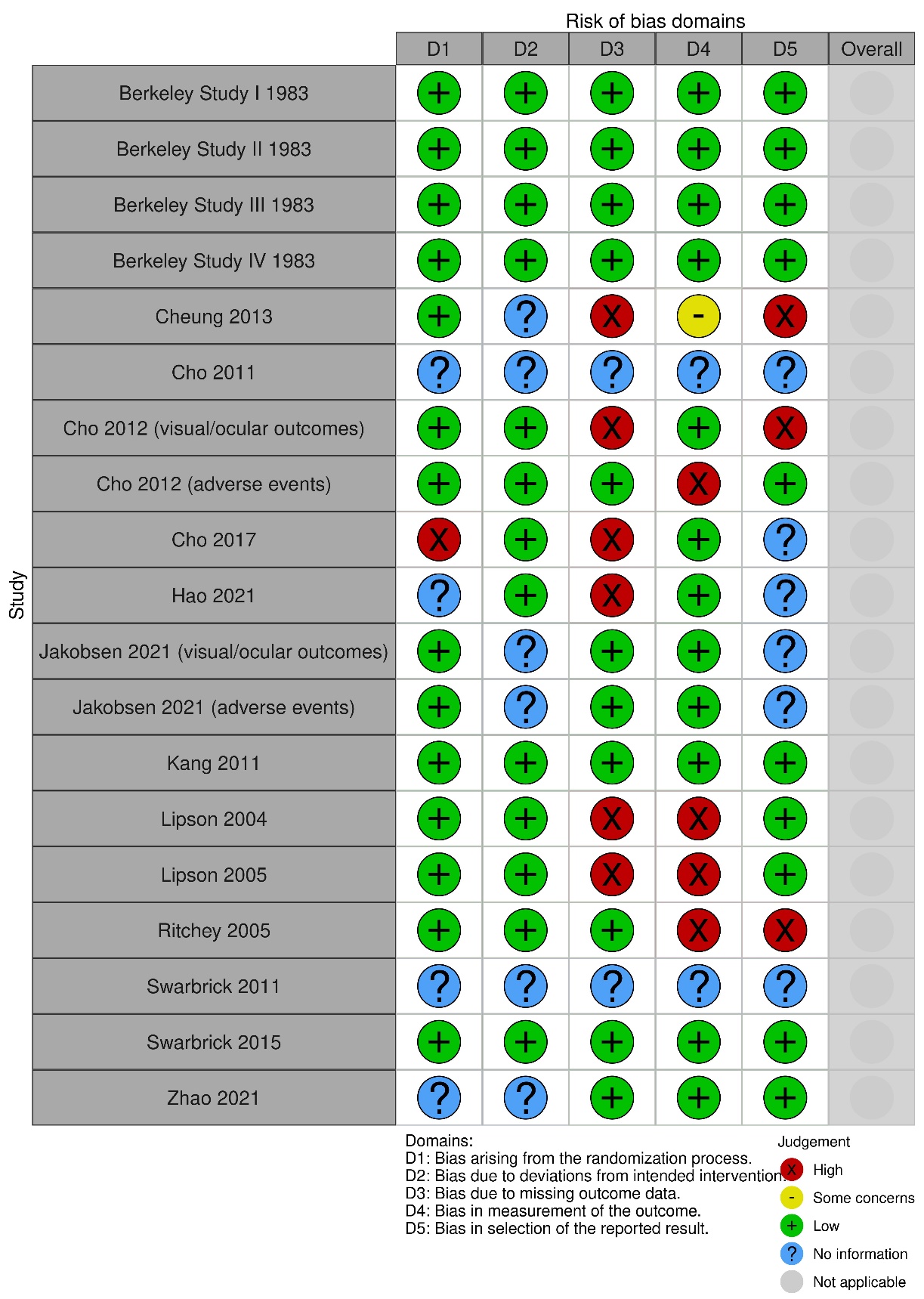


**Figure 1. Risk of bias in randomized-controlled trials (Visual and ocular biometric and adverse events combined).** Risk of bias was assessed using the Cochrane Risk of Bias tool [5]. Overall risk of bias is not assessed for RCTs and thus this column remains empty.

# **Risk of bias in non-randomized studies**

Risk of bias for n = 24 non-randomized studies reporting visual and ocular biometric outcomes is summarized in Figure 2c of main paper, with detailed results reported in Figure 2. Risk of bias for n = 16 non-randomized studies reported on adverse outcomes is summarized in Figure 2d of main paper, with detailed results reported in Figure 3. Studies with critical risk of bias were excluded from data analysis.

Amongst studies reporting on visual and ocular biometrics: Overall risk of bias was low in n = 2 included studies, as only the MCOS study [15, 16] had a published study protocol. Other papers had an overall moderate (n = 3), serious (n = 17), or insufficient information to judge risk of bias (NI; n = 3). Ratings on individual domains for included studies are as follows.

Bias due to confounding was serious or critical in more than half of the studies (n = 7 low, n = 11 moderate, n = 7 serious). Multiple studies had baseline confounding due to baseline differences between groups, which is due to: differences in age between treatment and comparator groups [17-19], differences in baseline myopia [19] and differences in follow-up post-treatment [19]. One study recruited participants from a previous study and was deemed to have a serious risk of bias as success of treatment in the prior study may in turn have affected participants decision to participate in the subsequent study [20]. Two studies measuring quality of life only included participants that continued treatment which likely resulted in inherent bias towards those with a sufficiently high quality of life to continue their treatment [21, 22].

Bias due to selection of participants was low in n = 11 studies, moderate in n = 3, serious in n = 6 and NI in n = 6. The most typical problem is selection of participants on the basis of outcomes reported after treatment, such as satisfactory visual outcomes being achieved by participants [21, 23]. Exclusion of participants who discontinued treatment was a concern for multiple studies, as it was typically unclear if these participants discontinued due to poor response to treatment [18, 24-26], and may have resulted in outcomes in those continuing treatment being positively biased, particularly in longer term studies. While most studies were conducted with bilateral OK use, others included only unilateral myopic patients, and excluded participants for which the emmetropic eye developed myopia during the study [24]. These selection biases typically also introduced confounding to the studies.

Bias due to classification of interventions was typically low (n = 21), as it generally was unambiguous which treatment participants were given based on group allocation or prescription records for participant files. However, n = 3 studies still had a moderate risk of bias, and n = 2 studies provided insufficient information on methods used to classify each intervention in order to judge risk of bias (NI).

Bias due to deviation from intended interventions was also typically low (n = 11) and moderate in n = 5 studies. N = 5 studies were rated NI, with these studies typically not reporting adherence to treatment and length of discontinuations (if any) in response to participant adverse events.

Bias due to missing data was mostly low in (n = 16), moderate in n = 4, and serious in n = 2. Reasons for serious risk of bias included inadequate reporting of data for the comparator group [27] or both groups [28]. n = 2 studies provided insufficient information for judgement in this domain, typically due to underreporting of collected outcomes in methods.

Bias in measurement of outcomes was low in n = 17 studies, moderate in n = 4, serious in n = 4 and NI in n = 1 papers. Serious risk of bias was inherent to the studies which examined quality of life (e.g. [21, 22, 29]). As there was high prevalence of automatic measuring biometric outcomes, low risk of bias was however common, although as manual measurements with necessary in studies without this equipment, the lack of masking for outcome assessors resulted in a serious risk of bias [30].

Bias in selection of reported result was low in n = 8 studies, moderate in n = 10 studies, serious in n = 5 studies, and NI in n = 3 studies. Two of the MCOS papers had a low risk of bias [15, 16] as they made reference to a published protocol. Of the majority of papers with a moderate risk of bias, reporting typically appeared to be relatively thorough, but this could not be confirmed due to the lack of published protocols or analysis plans. Of those with a serious risk of bias, judgement was on the basis of lack of reporting of numerical data for the comparator group [27], failing to report outcomes for each time point [20], and failing to report outcomes in both eyes (or provide analysis demonstrating outcomes did not differ between eyes) when this data was collected [31] Of the one study rated NI, this was on the basis that such limited information on outcomes recorded was present in the protocol it was difficult to judge the completeness of reporting of data [32].

Amongst studies reporting on adverse events, n = 4 included studies had moderate ratings, n = 9 serious risk of bias, n = 3 provided insufficient information on one or more domains to accurately classify overall risk of bias (NI), and no study had a low risk of bias.

Bias due to confounding was low in n = 6 studies, moderate in n = 5 and serious in n = 5 studies. Baseline differences between groups contributed to confounding in many studies to some degree. Examples in confounding factors included variability in prior experience with contact lens wear within or between groups [20, 33], different availability of data between treatment groups (OK patients having more visits and thus more available data) [28, 34], and differences in mean age at treatment commencement between each treatment group [18, 28, 35]. In some studies [33] there were also differences in study outcomes at baseline, in addition to differences in contact lens wear experience, with all soft contact lens (SCL) but no OK wearers having worn their treatment modality previously, and indeed 30% of OK wearers never having worn contact lenses at all prior. The degree of confounding was often difficult to assess in hospital audit studies however, given these were retrospective studies with a wide range of contact lenses and contact lens solutions worn by patients of a wide range of ages, and for differing lengths of time.

Bias due to selection of participants was low in n = 6 studies, moderate in n = 5, serious in n = 3. There were n = 2 studies rated NI, with insufficient information to assess risk of bias due to no information being provided regarding participant inclusion and exclusion criteria [36] or the study including both newly recruited participants and participants from a previous study, for which no details being provided regarding the latter [20]. However, the studies with serious risk of selection bias were those which chose to only include participants who had completed the full-length duration of their studies [18, 37], and thus those which may have discontinued treatment due to facts associated with adverse events were excluded from the study and were not reported upon. Hospital audit studies typically had some unavoidable degree of selection bias, as these studies retrieved data from cases of microbial or acanthamoeba keratitis cases from hospital records, and thus included only participants severe enough to report to the hospital [eg. 38, 39]. [18, 37]. The only study of this format with a serious risk of bias however was judged so on the basis that only included participants with unilateral infection were included in the study [34].

Most included papers had a low bias due to classification of interventions (n = 12), since most of the studies had a systematic approach in allocating or recording interventions status as outlined in their methods. Retrospective studies most typically relied on records regarding what lenses were prescribed to participants in their patient records. Only the study by Garcia-Porta and colleagues had a rating of moderate in this domain, specifically because of participants choosing their own treatment, which may have been affected by knowledge or risk of the outcomes of interest. Specifically, the treatment allocation process reported to be based on “personal preferences and taking into account whether their ocular parameters allowed correcting the full myopia with the OK treatment or did not”[33]. Additionally, n = 3 hospital audit studies provided insufficient information to judge risk of bias in this domain, as this aspect of their methods was sparsely detailed. No included studies had a serious rating on this domain.

The majority of studies had low bias of deviations from intended exposure (n = 10) and n = 2 studies did not provide sufficient information in this domain [37]. N = 1 study had a moderate risk of bias as participants that experienced adverse events discontinued treatment for variable durations, which while medically appropriate meant that participants wore their treatment modality for different lengths of time overall [20]. Amongst the n = 3 studies with serious risk of bias, 2 has this rating because of participants drop-out after dissatisfaction with intervention [33, 36]. An additional n = 1 study had a serious risk of bias on this domain because participants were reported to wash their OK lenses with tap water or wear them in the shower [35], and thus this did not necessarily represent typical contact lens wear but rather outliers in behavior that resulted in infection.

Bias due to missing data was typically low, in n = 12 studies, and n = 1 study was rated as having a moderate risk of bias, and n = 1 was rated NI. N = 2 studies were judged to have a serious risk of bias ratings. In the first study it was due to the exclusion of data from participants with short follow-up (<3 months) to “prevent improper extrapolation of progression rates”, combined with a majority of participants in the advice/no-treatment control group rarely followed for longer than 3 months [28]. In the second study data regarding corneal outcomes was only reported for the historical control group, and it was unclear whether this data was absent from p or if it was not collected [40].

Bias in measurement of outcomes was low in n = 7 studies, moderate in n = 2 studies, serious in n = 2 studies and lacking in sufficient information for judgement in n = 5 studies. The serious risk of bias in the studies examining Dry Eye specifically was due to use of the Dry Eye Questionnaire [33, 36], as use of participant self-reported tools inherently results in a serious risk of bias rating.

Bias in selection of reported result was typically moderate (n = 9), with n = 4 papers having low risk of bias and n = 3 papers rated as having a serious risk of bias. Of the three studies with a serious risk of bias, the study by Turnbull and colleagues was rated as serious because an atropine treated group was reported in the methods but no data regarding this group was reported in the results [28]. Hiraoka and colleagues had introduced confounding by having a portion of participants in their study recruited from a previous study, but no data on this subgroup compared to newly recruited participants was reported [20]. Additionally, there was no true baseline data for OK patients, but rather axial length after 3 months of treatment, and while there were three-monthly follow-ups, data was only reported for each year [20]. Finally, the LORIC study did not adequately report corneal staining as an outcome for the control group [40].


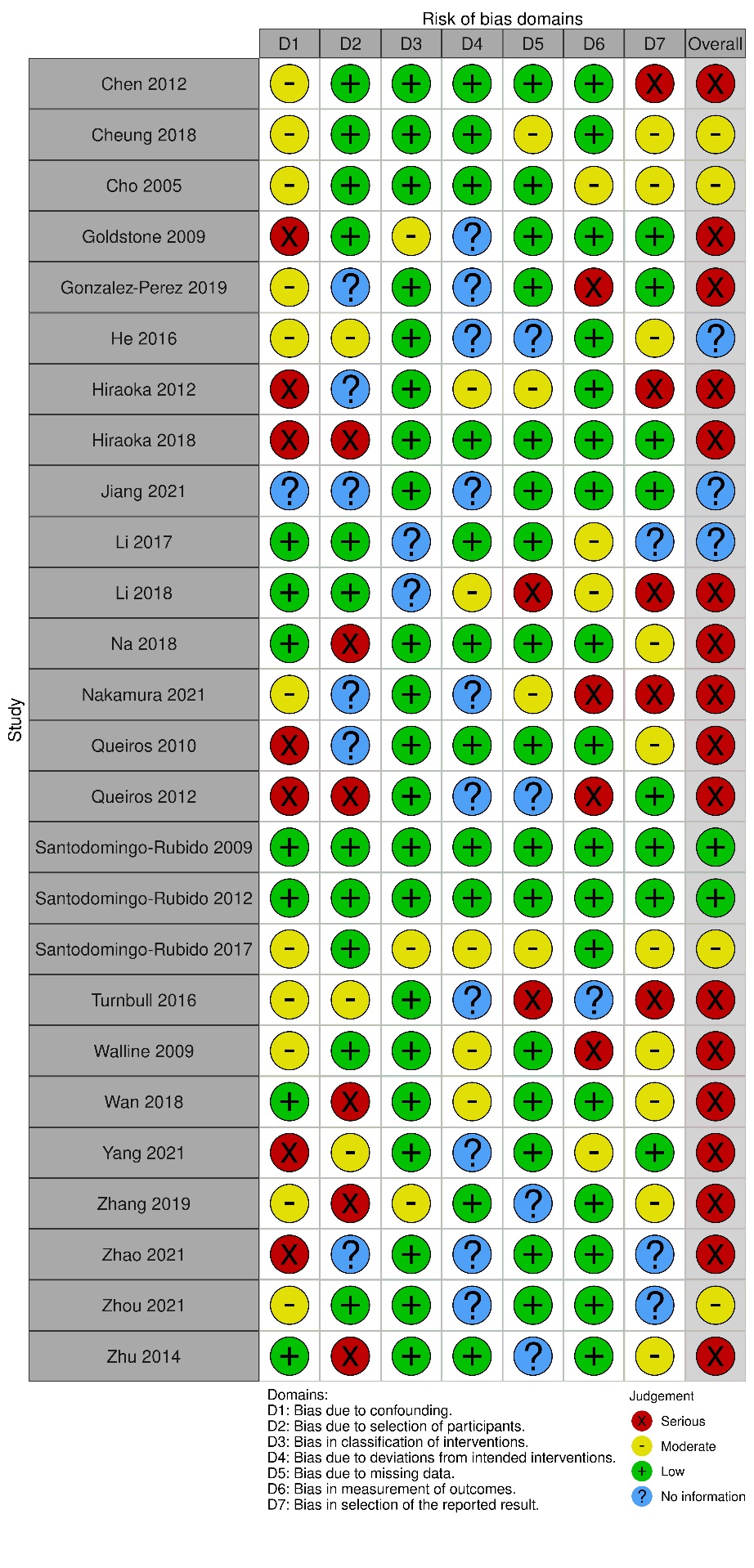
**Figure 2. Risk of bias in non-randomized controlled trials reported on visual and ocular biometrics.** Risk of bias was assessed using the Cochrane Risk of Bias for Non-Randomized Studies tool (ROBINS-I).


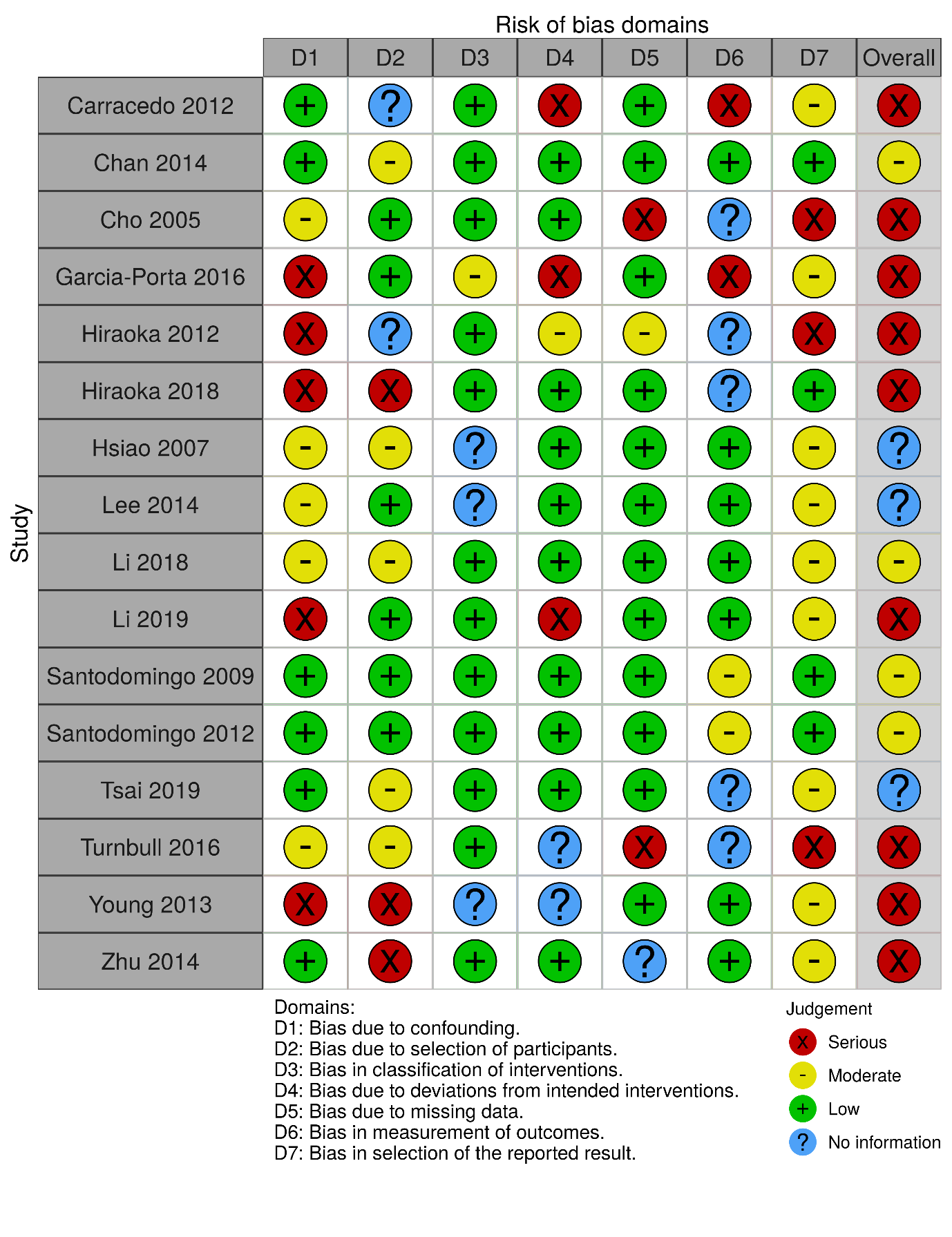


**Figure 3. Risk of bias in non-randomized studies reported on adverse events.** Risk of bias was assessed using the Cochrane Risk of Bias for Non-Randomized Studies tool (ROBINS-I).

**Supplementary Document 3. Summary of adverse events.**

*Adverse events: Incidence*

Incidence of adverse events was reported in four studies (n = 657 participants) [9, 18, 41, 42], though due to inconsistencies in reporting they were unable to be compared using meta-analysis. The results are summarised as follows.

Lipson et al., [9] reported on the percentage of adult participants found to have an adverse event at follow-up visits combined. The complications reported were hyperaemia, oedema, superficial punctate keratopathy (SPK) and iron deposition. The events were reported as the number of patients with an adverse event compared with the total number of visits; in the OK group 55 patients over 203 visits were found to have an adverse event and in the SCL comparator group 53 patients over 150 visits were found to have an adverse event.

Hirakoa et al., [18] reported the incidence of adverse events in children (8-16 years) as a percentage of eyes per annum (10 years of retrospective records), where recurrences of the same complication in an eye was classed as different event. These are reported as the number of eyes with an adverse event over the total number of eyes in the study, where in the OK group 119 events were seen in 53 eyes and 0 events in 51 eyes and in the SCL comparator group 103 events were seen in 43 eyes and 0 events in 43 eyes. The events reported included allergic, phlyctenular conjunctivitis, SPK, corneal erosion, corneal infiltration, blepharitis, hordeolum and chalazion.

Santodomingo-Rubido et al., [42] reported on adverse events in children (6-12 years) against a spectacle wearing comparator group as a percentage of eyes per annum. This study was thorough in its classification of events into non-significant, significant, and serious complications in addition to grading the severity of complications, such as corneal staining. A greater incidence of adverse events was seen in the ortho-K group, with 16 adverse events in 31 participants, compared to none in the spectacle comparator group of 24 participants, corresponding to 0% serious events, 5.8% significant events and 9.2% non-significant events in the treatment group.

The Berkeley study [41] reported the percentage of adult participants with corneal oedema, central and limbal staining and the corresponding grade (1-3) which was pooled value from all eye examinations over all visits, separately for morning (AM) and afternoon (PM) examinations. The total number of follow-up visits for review of complications was also reported, of which there were 76 for the ortho-K group (n = 31 participants), and 56 for the comparator group (n = 28 participants).

*Corneal staining*

Corneal staining was measured differently by the various studies that examined this outcome. It was not possible to directly perform subgroup analysis with paediatric and adult patients, as all corneal staining was measured differently in studies with adults and children. The number of participants with corneal staining was reported in 5 studies with paediatric participants [13, 26, 40, 42, 43] each with spectacles as the comparator group. Participants wearing orthokeratology lenses more frequently experienced this adverse event, with n = 32 ortho-K wearing participants reporting corneal staining compared to n = 0 participants in the comparator groups (OR 17.55, 95% CI 4.23 to 72.81; participants = 360). This is unsurprising however given the comparator group in all four studies wore spectacles.

The Cornea and Contact Lens Research Unit grading scales (CCLRU) were used to report corneal staining in n = 2 studies with adult participants [33, 36]. The corneal staining grade was lower at 1 month in ortho-K wearers than in the soft or rigid contact lens wearing comparator groups (MD 0.37, 95% CI 0.14 to 0.61; participants = 98).  García-Porta and colleagues reported outcomes after 3 months of wear [33] and observed that ortho-K staining grade did not differ from soft-contact lens wearers after 3 months of wear (ortho-K: 0.62 ± 0.62, SCL: 0.64 ± 0.56). Corneal staining depth scores also did not differ between orthokeratology and a soft contact lens comparator group at either 1 month (ortho-K: 0.63 ± 0.77; SCL: 0.75 ± 0.72) or at 3 months of treatment in either the morning (ortho-K: 0.62 ± 0.62; SCL: 0.64 ± 0.56) or afternoon (ortho-K: 0.50 ± 0.58; SCL: 0.54 ± 0.58).

The Berkeley study reported incidence of central corneal and limbal staining in adult participants [41]. Both the ortho-K and RGP comparator lenses were worn during the day for this study, and data was collected for corneal staining in the morning and evening. For morning appointments, in ortho-K wearing participants there was a 26.6% incidence of Grade 1 and 0.2% incidence of Grade 2 central corneal staining, and a 47.7% incidence of Grade 1 and 0% of Grade 2 or higher incidence of limbal staining. For evening appointments, of which there was a 22% incidence of Grade 1 and 0 % incidence of central corneal staining and 59.4% incidence of Grade 1 and 0% incidence of Grade 2 or higher limbal staining in orthokeratology wearing eyes (n = 610 eye measurements in n = 31 participants) after 12 months of wear. By comparison, there was an 18.7% incidence of Grade 1 and 0% incidence of Grade 2 or higher central corneal staining and 59.9% incidence of Grade1 and 0% incidence of Grade 2 or higher limbal staining in the rigid contact lens wearing comparator group (n = 516 eye measurements in n = 28 participants).

Jakobsen et al., [43] report corneal staining of grade 3 depth occurring in 2 OK participants and none, though they do not differentiate between grades 1-2.

*Dimple veiling*

This was a rare adverse event, and thus was rarely reported in any studies. The MCOS study was the only study to record this (n = 53 participants) [44] and reported that dimple veiling occurred in only one orthokeratology wearing participant in the entire study and did not occur in the spectacle-wearing comparison group.

*Dry eye*

Dry eye symptoms were reported in n = 98 eyes by 2 studies after 1 month [33, 36] or 3 months of treatment [33], with comparator groups being either soft or rigid contact lenses. The outcomes reported include morning tear volume, and discomfort and dryness Dry Eye Questionnaires (DEQ) scores. Both studies reported outcomes in adults only. García-Porta and colleagues additionally reported DEQ scores during the first two hours of wear and end of the day, and collected tear volume at these two periods [33]. Accordingly, for the three month timepoint we present this data as the more clinically relevant timepoints “after period of contact lens wear” (first two hours of the day for ortho-K and end of the end for SCL) and “after non-wear period” (afternoon for ortho-K and first two hours of the day for SCL).

Tear volume

After 1 month of wear, morning tear volume, measured by Schirmer tests, tended to be higher in orthokeratology wearing participants compared to non-orthokeratology wearers (MD 4.32 mm, 95% CI -0.06 to 8.70). This was true for ortho-K compared to both RGP (ortho-K: 21.8 ± 13.18 mm, RGP: 14.5 mm ± 9.38 mm, [36]) and SCL (ortho-K: 21.09 ± 11.44, SCL: 18.93 ± 9.50, [33].

Tear volumes after the period of contact lens wear was 20.73 ± 11.1 mm in orthokeratology (tears collected in the morning) and 19.03 ± 11.05 mm in soft contact lens users (tears collected in the afternoon). When tears were collected after the non-wear period, tear volume was 18.32 ± 11.81 mm for orthokeratology (tears collected in the afternoon) and 21 ± 11.79 mm in soft contact lens comparator group (tears collected in the morning).

DEQ dryness score

There was no difference in DEQ dryness scores between orthokeratology and other treatment modalities in the morning after one month of treatment (MD 0.26, 95% CI -0.62 to 0.10). Scores differed between treatment modalities, with scores being higher in ortho-K compared to RGP (ortho-K: 1.5 ± 1.08, RGP: 1.05 ± 0.84, [36]), but lower in ortho-K compared to SCL (ortho-K: 0.87 ± 0.87, SCL: 1.58 ± 0.81 [33]).

Garcia-Porta and colleagues recorded that after three months of treatment the overall dryness scores were 1.27 ± 0.88 for the orthokeratology group and 1.43 ± 0.77 for SCL. After the wear period, dryness scores were 1.00 ± 1.15 for the orthokeratology group and 1.90 ± 1.18 for the SCL comparator group. After the non-wear period dryness scores were 1.14 ± 1.08 for ortho-K wearers and 0.57 ± 0.63 for the soft contact lens comparator group.

DEQ discomfort score

There was no difference between treatments in DEQ discomfort score after 1 month of treatment (MD 0.06, 95% CI -0.28 to 0.39). Scores differed between treatment modalities, with scores being higher in ortho-K compared to RGP (ortho-K: 1.9 ± 1.17, RGP: 0.95 ± 0.61, [36]), but lower in ortho-K compared to SCL (ortho-K: 0.96 ± 0.82, SCL: 1.45 ± 0.77 [33]).

After 3 months of treatment, Garcia-Porta and colleagues reported that overall discomfort scores were 0.92 ± 0.81 for the orthokeratology group and 1.17 ± 0.63 for SCL. After the contact lens wear period discomfort scores were 0.82 ± 1.14 for the orthokeratology group and 1.67 ± 1.09 for the SCL group. After the non-wear period, discomfort scores were 0.77 ± 0.92 for the orthokeratology group and 0.73 ± 0.83 for SCL.

*Epithelial iron deposition*

Lipson and colleagues [45] reported the incidence of this outcome in n = 108 eyes, and had a 2.95% incidence in ortho-K wearing eyes (6 out of 203 visits) compared to 0% incidence in SCL wearing eyes (0 out of 150 visits).

*Lens binding*

This outcome was reported in n = 2 studies [28, 46], each with paediatric participants. It was a rare adverse event, instance of which did not differ between orthokeratology wearing and contact lens wearing comparator groups (OR 0.32, 95% CI 0.01 to 8.24; participants = 84). While Turnbull and colleagues did not record any event of lens binding for either ortho-K or SCL treatment, Swarbrick and colleagues reported lens binding occurred in n = 1 RGP wearer, but did not occur to any participants in the ortho-K wearing group.

*Corneal infiltrates*

Corneal infiltration and keratitis was reported by only 1 study (n = 182 participants) [18]. After 10 years of therapy, there was a higher incidence in the ortho-K group, with a total incidence of 8 cases of infiltrates reported (0.8% of eyes per annum), compared to 0 cases reported in the SCL comparator group. None of the infiltrates were deemed to be microbial keratitis. No other studies reported on keratitis as an adverse event.

**References**

[1] Guyatt GH, Oxman AD, Schünemann HJ, Tugwell P, Knottnerus A. GRADE guidelines: A new series of articles in the Journal of Clinical Epidemiology. Journal of Clinical Epidemiology 2011;64(4):380-2. <https://doi.org/https://doi.org/10.1016/j.jclinepi.2010.09.011>.

[2] Innovation VH. Covidence systematic review software. Melbourne, Australia: Veritas Health Innovation.

[3] Australia Co. How to use the evidence: assessment and application of scientific evidence. Handbook series on preparing clinical practice guidelines. In: Council NHaMR, ed. Canberra: Commonwealth of Australia; 2000.

[4] Collaboration TC. Review Manager (RevMan). Version 5.3. ed. Copenhagen: Nordic Cochrane Centre; 2014.

[5] Sterne JAC, Savović J, Page MJ, Elbers RG, Blencowe NS, Boutron I, et al. RoB 2: a revised tool for assessing risk of bias in randomised trials. Bmj 2019;366:l4898. <https://doi.org/10.1136/bmj.l4898>.

[6] Sterne J, Hernán M, Reeves B, Savović J, Berkman N, Viswanathan M, et al. ROBINS-I: a tool for assessing risk of bias in non-randomized studies of interventions. BMJ 2016;355:i4919. <https://doi.org/10.1136/bmj.i4919>.

[7] Cho P, Cheung SW. Discontinuation of orthokeratology on eyeball elongation (DOEE). Cont Lens Anterior Eye 2017;40(2):82-7.

[8] Brand RJ, Polse KA, Schwalbe JS. The Berkeley Orthokeratology Study, Part I: General conduct of the study. Am J Optom Physiol Opt 1983;60(3):175-86.

[9] Lipson MJ, Sugar A, Musch DC. Overnight corneal reshaping versus soft daily wear: A visual quality of life study (interim results). Eye and Contact Lens 2004;30(4):214-7.

[10] Lipson MJ, Sugar A, Musch DC. Overnight corneal reshaping versus soft disposable contact lenses: vision-related quality-of-life differences from a randomized clinical trial. Optometry and vision science 2005;82(10):886‐91-‐91.

[11] Ritchey ER, Barr JT, Mitchell GL. The comparison of overnight lens modalities (COLM) Study. Eye and Contact Lens 2005;31(2):70-5.

[12] Cho P, Chan B, Cheung SW, Mountford J. Do fenestrations affect the performance of orthokeratology lenses? Optom Vis Sci 2012;89(4):401-10.

[13] Cho P, Cheung SW. Retardation of myopia in Orthokeratology (ROMIO) study: a 2-year randomized clinical trial. Invest Ophthalmol Vis Sci 2012;53(11):7077-85.

[14] Cheung SW, Cho P. Validity of axial length measurements for monitoring myopic progression in orthokeratology. Invest Ophthalmol Vis Sci 2013;54(3):1613-5.

[15] Santodomingo-Rubido J, Villa-Collar C, Gilmartin B, Gutiérrez-Ortega R. Myopia control with orthokeratology contact lenses in Spain (MCOS): study design and general baseline characteristics. Journal of Optometry 2009;2(4):215-22.

[16] Santodomingo-Rubido J, Villa-Collar C, Gilmartin B, Gutiérrez-Ortega R. Myopia control with orthokeratology contact lenses in Spain: refractive and biometric changes. Investigative ophthalmology & visual science 2012;53(8):5060‐5-‐5.

[17] Goldstone RN, Yildiz EH, Fan VC, Asbell PA. Changes in higher order wavefront aberrations after contact lens corneal refractive therapy and LASIK surgery. Journal of Refractive Surgery 2009;25(5):1-8.

[18] Hiraoka T, Sekine Y, Okamoto F, Mihashi T, Oshika T. Safety and efficacy following 10-years of overnight orthokeratology for myopia control. Ophthalmic Physiol Opt 2018;38(3):281-9. <https://doi.org/10.1111/opo.12460>.

[19] Queirós A, González-Méijome JM, Villa-Collar C, Gutierrez JR, Jorge J. Local steepening in peripheral corneal curvature after corneal refractive therapy and LASIK. Optometry and vision science : official publication of the American Academy of Optometry 2010;87(6):432-9.

[20] Hiraoka T, Kakita T, Okamoto F, Takahashi H, Oshika T. Long-term effect of overnight orthokeratology on axial length elongation in childhood myopia: A 5-year follow-up study. Investigative Ophthalmology and Visual Science 2012;53(7):3913-9.

[21] Queirós A, Villa-Collar C, Gutiérrez AR, Jorge J, González-Méijome JM. Quality of life of myopic subjects with different methods of visual correction using the NEI RQL-42 questionnaire. Eye and Contact Lens 2012;38(2):116-21.

[22] Yang B, Ma X, Liu L, Cho P. Vision-related quality of life of Chinese children undergoing orthokeratology treatment compared to single vision spectacles. Cont Lens Anterior Eye 2021;44(4):101350. <https://doi.org/10.1016/j.clae.2020.07.001>.

[23] Wan L, Wei CC, Chen CS, Chang CY, Lin CJ, Chen JJ, et al. The synergistic effects of orthokeratology and atropine in slowing the progression of myopia. J Clin Med 2018;7(9).

[24] Na M, Yoo A. The effect of orthokeratology on axial length elongation in children with myopia: Contralateral comparison study. Japanese journal of ophthalmology 2018;62(3):327-34.

[25] Zhang Y, Chen Y. Effect of orthokeratology on axial length elongation in anisomyopic children. Optometry and vision science : official publication of the American Academy of Optometry 2019;96(1):43-7.

[26] Zhu MJ, Feng HY, He XG, Zou HD, Zhu JF. The control effect of orthokeratology on axial length elongation in Chinese children with myopia. BMC ophthalmology 2014;14:141-.

[27] Li Z, Hu Y, Cui D, Long W, He M, Yang X. Change in subfoveal choroidal thickness secondary to orthokeratology and its cessation: a predictor for the change in axial length. Acta ophthalmologica 2018.

[28] Turnbull PR, Munro OJ, Phillips JR. Contact lens methods for clinical myopia control. Optometry and vision science : official publication of the American Academy of Optometry 2016;93(9):1120-6.

[29] González-Pérez J, Sánchez-García Á, Villa-Collar C. Vision-specific quality of life: laser-assisted in situ keratomileusis versus overnight contact lens wear. Eye & contact lens 2019;45(1):34-9.

[30] Walline JJ, Jones LA, Sinnott LT. Corneal reshaping and myopia progression. British Journal of Ophthalmology 2009;93(9):1181-5.

[31] Chen Z, Niu L, Xue F, Qu X, Zhou Z, Zhou X, et al. Impact of pupil diameter on axial growth in orthokeratology. Optometry and vision science : official publication of the American Academy of Optometry 2012;89(11):1636-40.

[32] Li Z, Cui D, Hu Y, Ao S, Zeng J, Yang X. Choroidal thickness and axial length changes in myopic children treated with orthokeratology. Contact Lens and Anterior Eye 2017;40(6):417-23.

[33] García-Porta N, Rico-del-Viejo L, Martin-Gil A, Carracedo G, Pintor J, González-Méijome JM. Differences in dry eye questionnaire symptoms in two different modalities of contact lens wear: Silicone-hydrogel in daily wear basis and overnight orthokeratology. Biomed research international 2016;2016.

[34] Young AL, Leung KS, Tsim N, Hui M, Jhanji V. Risk factors, microbiological profile, and treatment outcomes of pediatric microbial keratitis in a tertiary care hospital in Hong Kong. American Journal of Ophthalmology 2013;156(5):1040-4.e2.

[35] Li W, Wang Z, Qu J, Zhang Y, Sun X. Acanthamoeba keratitis related to contact lens use in a tertiary hospital in China. BMC Ophthalmol 2019;19(1):202-.

[36] Carracedo G, González-Méijome JM, Pintor J. Changes in diadenosine polyphosphates during alignment-fit and orthokeratology rigid gas permeable lens wear. Investigative Ophthalmology and Visual Science 2012;53(8):4426-32.

[37] Zhu M, Feng H, Zhu J, Qu X. The impact of amplitude of accommodation on controlling the development of myopia in orthokeratology. [Zhonghua yan ke za zhi] Chinese journal of ophthalmology 2014;50(1):14-9.

[38] Chan TCY, Li EYM, Wong VWY, Jhanji V. Orthokeratology-associated infectious keratitis in a tertiary care eye hospital in Hong Kong. American Journal of Ophthalmology 2014;158(6):1130-5.e2.

[39] Li W, Sun X, Wang Z, Zhang Y. A survey of contact lens-related complications in a tertiary hospital in China. Contact Lens and Anterior Eye 2018;41(2):201-4.

[40] Cho P, Cheung SW, Edwards M. The longitudinal orthokeratology research in children (LORIC) in Hong Kong: A pilot study on refractive changes and myopic control. Current eye research 2005;30(1):71-80.

[41] Polse KA, Brand RJ, Keener RJ, Schwalbe JS, Vastine DW. The Berkeley Orthokeratology Study, part III: safety. Am J Optom Physiol Opt 1983;60(4):321-8.

[42] Santodomingo-Rubido J, Villa-Collar C, Gilmartin B, Gutiérrez-Ortega R. Orthokeratology vs. spectacles: adverse events and discontinuations. Optom Vis Sci 2012;89(8):1133-9.

[43] Jakobsen TM, Møller F. Control of myopia using orthokeratology lenses in Scandinavian children aged 6 to 12 years. Eighteen‐month data from the Danish Randomized Study: Clinical study Of Near‐sightedness; TReatment with Orthokeratology Lenses (CONTROL study). Acta ophthalmologica (Oxford, England) 2022;100(2):175-82. <https://doi.org/10.1111/aos.14911>.

[44] Santodomingo-Rubido J, Villa-Collar C, Gilmartin B, Gutiérrez-Ortega R. Myopia control with orthokeratology contact lenses in Spain (MCOS): predictive factors associated with myopia progression. Contact lens & anterior eye 2012;35:e16-e.

[45] Lipson MJ. Long-term clinical outcomes for overnight corneal reshaping in children and adults. Eye and Contact Lens 2008;34(2):94-9.

[46] Swarbrick HA, Alharbi A, Watt K, Lum E, Kang P. Myopia control during orthokeratology lens wear in children using a novel study design. Ophthalmology 2015;122(3):620-30.
