# Supplementary material for: Benefits and risks of orthokeratology treatment: a systematic review and meta-analysis
Source: Int Ophthalmol. 2024 Jun 21;44(1):239. doi: 10.1007/s10792-024-03175-w (PMC11192849; doi:10.1007/s10792-024-03175-w)
Supplement: Supplementary file 2 — Supplementary file2 (DOCX 286 KB) [file 10792_2024_3175_MOESM2_ESM.docx]

International Ophthalmology

**Benefits and risks of orthokeratology treatment – a systematic review and meta-analysis**

Lauren Sartor* MChD,^2,3^ Damien S. Hunter* PhD,^1,2^ Mai Linh Vo* MD,^2^ Chameen Samarawickrama FRANZCO ^1,2,3^

^1^Centre for Vision Research, Westmead Institute of Medical Research, Sydney, Australia

^2^Faculty of Medicine and Health, University of Sydney, New South Wales, Australia

^3^Department of Ophthalmology, Westmead Hospital, New South Wales, Australia

**Corresponding Author:**

Associate Professor Chameen Samarawickrama

Email: chameen.sams@sydney.edu.au

**Contents**

Supplementary Figure 1a-d. Funnel plots for sensitivity analyses of change in axial length 3

Supplementary Figure 2. Funnel plot for number of participants experiencing adverse events

in orthokeratology vs comparator treatments 4

Supplementary Table 1: Characteristics of included randomized controlled trials 5

Supplementary Table 2: Characteristics of included non-randomized studies 10

Supplementary Table 3: Change in axial length in orthokeratology (OK) vs comparator treatments 17

Supplementary Table 4: Sensitivity analyses 19

Supplementary Table 5: Axial length in orthokeratology (OK) vs comparator treatments 22

Supplementary Table 6: Change in axial length following discontinuation of orthokeratology (OK) vs

ongoing OK treatment, or treatment prior to discontinuation 23

Supplementary Table 7: Spherical equivalent refractive error in orthokeratology (OK) vs

comparator treatments. 24

Supplementary Table 8: Corneal measurements in orthokeratology (OK) vs comparator treatments 25

Supplementary Table 9: Patient satisfaction in orthokeratology (OK) vs comparator treatments 26

Supplementary Table 10. Number of participants experiencing adverse events 27

Supplementary Table 11: Incidence of adverse events between treatment groups 29

Supplementary Table 12: Incidence and severity of dry eye symptoms in orthokeratology (OK)

vs comparator treatments 32


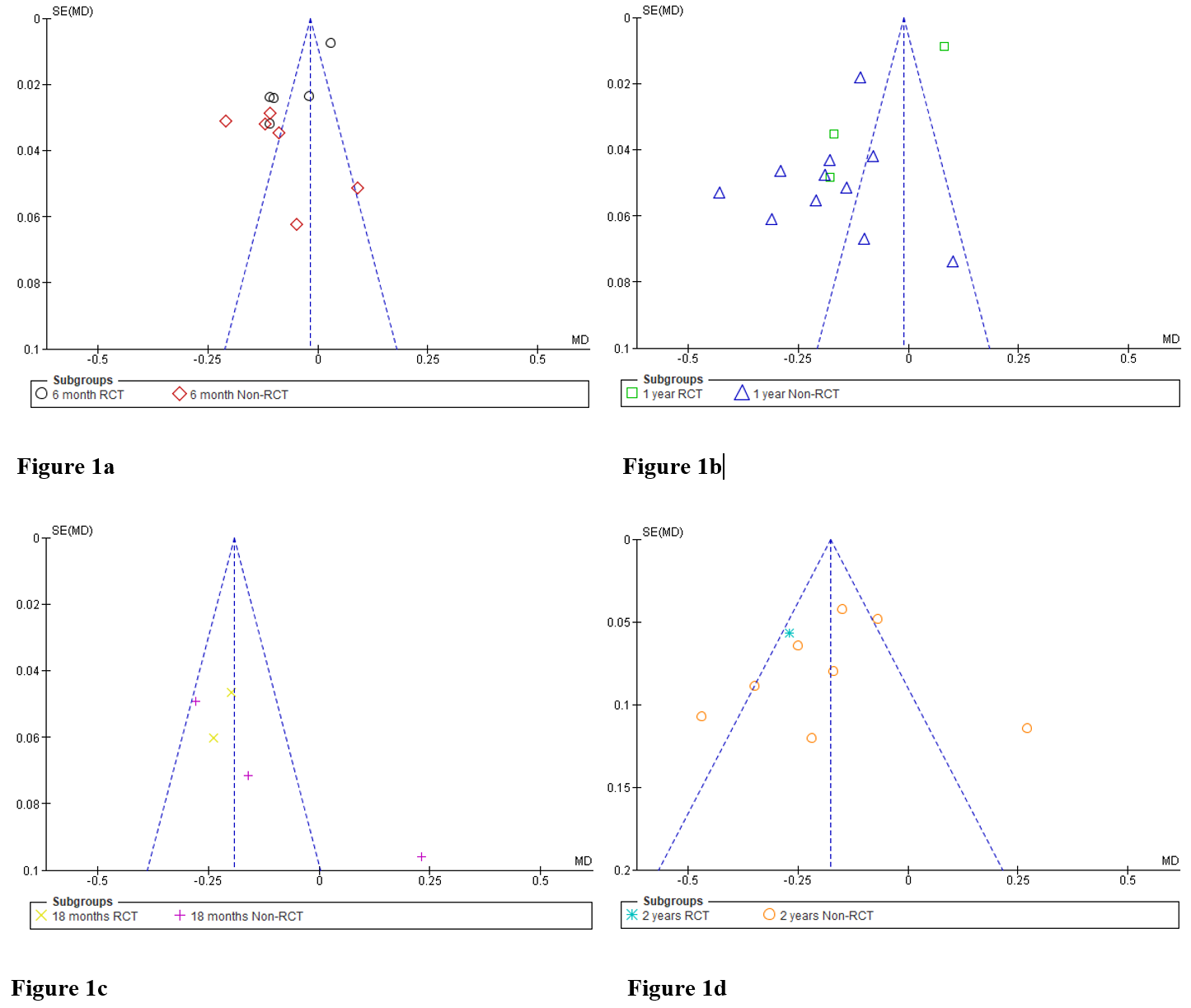


**Supplementary Figure 1a-d. Funnel plots for sensitivity analyses of change in axial length after 6, 12, 18 or 24 months of orthokeratology or comparator treatment**. Subgroups within Chen 2012 [1] and Zhu 2014 [2] are separately indicated within each funnel plot.

**References**

1. Chen Z, Niu L, Xue F, et al. Impact of pupil diameter on axial growth in orthokeratology. *Optometry and vision science : official publication of the American Academy of Optometry*. 2012;89(11):1636-1640.

2. Zhu MJ, Feng HY, He XG, Zou HD, Zhu JF. The control effect of orthokeratology on axial length elongation in Chinese children with myopia. *BMC ophthalmology*. 2014;14:141-141.


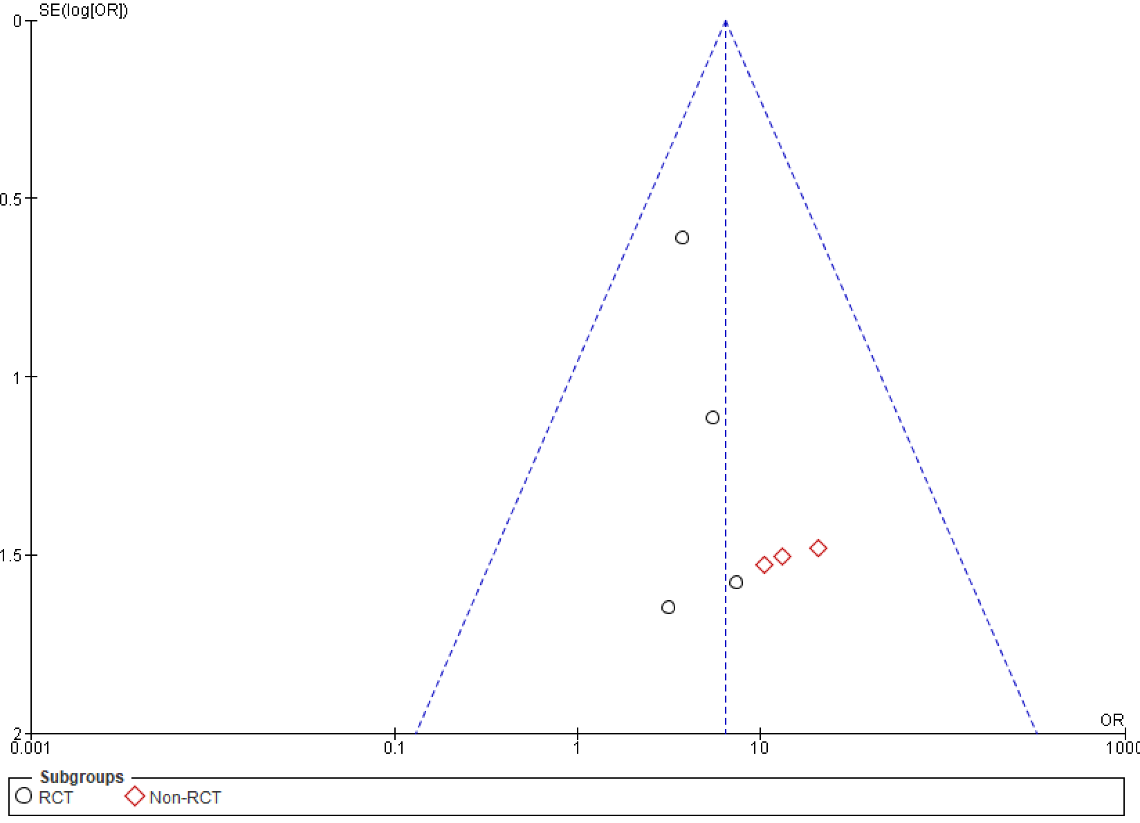


**Supplementary Figure 2. Funnel plot for number of participants experiencing adverse events in orthokeratology vs comparator treatments**. Studies are either categorized as randomized (RCTs) or non-randomised studies (non-RCTs)

# **Supplementary Table 1.** **Characteristics of included randomized controlled trials**

| **Study** | **Study design** | **Country** | **Age group** | **Sample size** | **Study duration** | **Ortho-K lens type** | **Comparator(s)** | **Data extracted** |
| --- | --- | --- | --- | --- | --- | --- | --- | --- |
| Berkeley Study (Brand 1983, Polse 1983)  [1-4] | RCT | USA | Adult | 80 | 1 year + 3 months discontinuation | Polycon | Conventional rigid contact lenses (Polycon) | Change in spherical equivalent refractive error, change in refractive cylinder, change in uncorrected visual acuity, change in best-corrected visual acuity, change in corneal curvature (horizontal), change in corneal curvature (vertical), change in central corneal thickness, persistent change in spherical equivalent refractive error, persistent change in uncorrected visual acuity, persistent change in corneal curvature (horizontal), persistent change in corneal curvature (vertical), adverse events, corneal staining |
| COLM study (Ritchey 2005) [5] | RCT | USA | Adult | 18 | 3 months | Paragon CRT (Paragon Vision Sciences, Inc., Mesa, AZ) | Soft contact lenses (Focus NIGHT & DAY, CIBA Vision) | Spherical refraction, high-contrast best-corrected visual acuity, low-contrast best-corrected visual acuity, corneal curvature (horizontal), changes in corneal curvature (vertical), change in uncorrected visual acuity, satisfaction with correction |
| DOEE (Cho 2017) [6] | RCT | Hong Kong | Children | 44 | 3 years and 2 months; or 2 years + 7 months discontinuation + 7 months ortho-K resumption | Menicon Z Night (Menicon Ltd) and Night Toric lenses (NKL Contactlenzen BV,Emmen, The Netherlands) | Single-vision spectacles (Founder Optical Company, Hong Kong), discontinuation | Change in axial length |
| Hao 2021 [7] | RCT | China | Children | 75 | 12 months | Euclid Systems Ortho-K; Euclid System Corp., Herndon, USA | 0.01% atropine by Shenyang Xingqi pharmaceutical company (Shenyang, China) and spectacles (brand not reported) | Change in axial length |
| Jakobsen 2021 [8] | RCT | Denmark | Children | 60 | 18 months | Dreamlite (Procornea, LZ Eerbeek, the Netherlands) | Single vision spectacles (Brands not reported) | Change in axial length, corneal staining |
| Kang 2011 [9] | RCT | Australia | Children | 16 | 3 months | BE or BE-A (Capricornia Contact Lens, Brisbane) | Rigid gas permeable contact lenses (J-Contour, Capricornia Contact Lens) | Change in spherical refraction, change in refractive cylinder, change in corneal curvature (flatter meridian), change in corneal curvature (steeper meridian) |
| ROMIO (Cho 2011, Cho 2012, Cheung 2013) [10-12] | RCT | Hong Kong | Children | 78 | 2 years | Menicon Z Night lenses (Menicon, Ltd) | Single-vision spectacles (Hong Kong Optical Lens Co, Hong Kong) | Change in axial length, uncorrected visual acuity, best-corrected visual acuity, adverse events, corneal staining |
| Swarbrick 2011, Swarbrick 2015 [13-14] | RCT | Australia | Children | 52 | 6 months + 6 months discontinuation | BE or A-BE (Capricornia Contact Lens Pty) | Soft contact lenses (J-Contour, Capricornia Contact Lens); discontinuation | Change in axial length, spherical equivalent refractive error, change in corneal curvature (flatter meridian), corneal curvature (steeper meridian), lens binding |
| Visual Quality of Life Study (Lipson 2004, Lipson 2005) [15-16] | RCT | USA | Children | 65 | 8 weeks | Paragon CRT (Paragon Vision Sciences, Inc., Mesa, AZ) | Soft contact lenses (Biomedics 55, Ocular Sciences, Concord, CA) | Uncorrected visual acuity, satisfaction with correction, adverse events, iron deposition |
| Zhao 2021 [17] | RCT | China | Children | 80 | 6-month and 1 year results | Euclid Systems OK; Euclid System Corp., Herndon, USA | Spectacles  (Atropine group and combination Atropine+OK not eligible for comparison) | Change in axial length, Uncorrected visual acuity, change in corneal curvature (flatter meridian), corneal curvature (steeper meridian) |

## **References**

1. Brand RJ, Polse KA, Schwalbe JS. The Berkeley Orthokeratology Study, Part I: General conduct of the study. *Am J Optom Physiol Opt*. 1983;60(3):175-186.

2. Polse KA, Brand RJ, Keener RJ, Schwalbe JS, Vastine DW. The Berkeley Orthokeratology Study, part III: safety. *Am J Optom Physiol Opt*. 1983;60(4):321-328.

3. Polse KA, Brand RJ, Schwalbe JS, Vastine DW, Keener RJ. The Berkeley Orthokeratology Study, Part II: Efficacy and duration. *Am J Optom Physiol Opt*. 1983;60(3):187-198.

4. Polse KA, Brand RJ, Vastine DW, Schwalbe JS. Corneal change accompanying orthokeratology. Plastic or elastic? Results of a randomized controlled clinical trial. *Arch Ophthalmol*. 1983;101(12):1873-1878.

5. Ritchey ER, Barr JT, Mitchell GL. The comparison of overnight lens modalities (COLM) Study. *Eye and Contact Lens*. 2005;31(2):70-75.

6. Cho P, Cheung SW. Discontinuation of orthokeratology on eyeball elongation (DOEE). *Cont Lens Anterior Eye*. 2017;40(2):82-87.

7. Hao Q, Zhao Q. Changes in subfoveal choroidal thickness in myopic children with 0.01% atropine, orthokeratology, or their combination. *Int Ophthalmol*. Sep 2021;41(9):2963-2971. doi:10.1007/s10792-021-01855-5

8. Jakobsen TM, Møller F. Control of myopia using orthokeratology lenses in Scandinavian children aged 6 to 12 years. Eighteen-month data from the Danish Randomized Study: Clinical study Of Near-sightedness; TReatment with Orthokeratology Lenses (CONTROL study). *Acta Ophthalmol*. Jul 7 2021;doi:10.1111/aos.14911

9. Kang P, Swarbrick H. Peripheral refraction in myopic children wearing orthokeratology and gas-permeable lenses. *Optometry and vision science : official publication of the American Academy of Optometry*. 2011;88(4):476-482.

10. Cheung SW, Cho P. Validity of axial length measurements for monitoring myopic progression in orthokeratology. *Invest Ophthalmol Vis Sci*. 2013;54(3):1613-1615.

11. Cho P, Cheung SW. Orthokeratology for slowing myopic progression: a randomised controlled trial. *Contact lens & anterior eye*. 2011;34:S2‐S3-S2‐S3.

12. Cho P, Cheung SW. Retardation of myopia in Orthokeratology (ROMIO) study: a 2-year randomized clinical trial. *Invest Ophthalmol Vis Sci*. 2012;53(11):7077-7085.

13. Swarbrick HA, Alharbi A, Lum E, Watt K. Overnight orthokeratology for myopia control: short-term effects on axial length and refractive error. *Contact lens and anterior eye*. 2011;34:S3-S3.

14. Swarbrick HA, Alharbi A, Watt K, Lum E, Kang P. Myopia control during orthokeratology lens wear in children using a novel study design. *Ophthalmology*. 2015;122(3):620-630.

15. Lipson MJ, Sugar A, Musch DC. Overnight corneal reshaping versus soft daily wear: A visual quality of life study (interim results). *Eye and Contact Lens*. 2004;30(4):214-217.

16. Lipson MJ, Sugar A, Musch DC. Overnight corneal reshaping versus soft disposable contact lenses: vision-related quality-of-life differences from a randomized clinical trial. *Optometry and vision science*. 2005;82(10):886‐891-886‐891.

17. Zhao Q, Hao Q. Clinical efficacy of 0.01% atropine in retarding the progression of myopia in children. Int Ophthalmol 2021;41(3):1011-7. https://doi.org/10.1007/s10792-020-01658-0.

# **Supplementary Table 2. Characteristics of included non-randomized studies, including hospital audits**

| **Study** | **Study design** | **Country** | **Age group** | **Sample size** | **Study duration** | **Ortho-K lens type** | **Comparator(s)** | **Primary data extracted** |
| --- | --- | --- | --- | --- | --- | --- | --- | --- |
| Carracedo 2012 [1] | Concurrent cohort study | Spain and Portugal | Adult | 22 | 1 month | Paragon CRT (Paragon Vision Sciences, Mesa, AZ, USA) | Rigid contact lenses (Oxicon HDS, Lenticon, Madrid, Spain) | Corneal staining, dryness, tear volume, discomfort |
| Chen 2012 [2] | Prospective cohort study | China | Child | 52 | 2 years | Boston XO (Hiline Optics, China) | Single-vision spectacles (Essilor, France) | Change in axial length |
| Cheung 2018 [3] | Prospective cohort study | Hong Kong | Child | 66 | 6 months | Menicon Z Night or Menicon Z Night Toric (NKL Contactlenzen BV, Emmen,The Netherlands) | Single-vision spectacles (Founder Optical Company, Hong Kong) | Change in axial length |
| Cho 2005 [4] | Historically controlled trial | Hong Kong | Child | 75 | 2 years | Boston XO or HDS 100 material (brand not reported) | Single-vision spectacles (brand not reported) | Change in axial length, change in spherical equivalent refractive error, change in corneal curvature (steeper meridian), change in corneal curvature (flatter meridian), corneal staining |
| García-Porta 2016 [5] | Concurrent cohort study | Portugal | Adult | 54 | 3 months | Paragon CRT or CRT Dual Axis (Paragon Vision Sciences, Mesa, AZ, USA) | Soft contact lenses (Biofinity, CooperVision, United States) | Frequency of discomfort, frequency of dryness, corneal staining type, corneal staining depth, tear volume |
| Goldstone 2009 [6] | Case-control study | USA | Adult | 23 | 1 month | Not reported | LASIK | Uncorrected visual acuity, spherical equivalent refractive error |
| González Pérez 2019 [7] | Prospective cohort study | Spain | Adult | 72 | 1 year | Paragon CRT 100 (Paragon Vision Sciences, Mesa, AZ, USA) | Soft contact lenses (Lotrafilcon A silicone-hydrogel, Alcon Pharmaceuticals Ltd,Fribourg, Switzerland); LASIK; non-myopic | Satisfaction with correction, overall NEI RQL-42 score |
| He 2016 [8] | Case-control study | China | Child | 271 | 1 year | Ortho-K LK Lens (Lucid, Korea) | Single-vision spectacles (brand not reported) | Change in axial length, axial length |
| Hiraoka 2012 [9] | Prospective cohort study | Japan | Child | 43 | 5 years | Emerald Lenses (Euclid Systems Corp., Herndon, VA) | Single-vision spectacles (brand not reported) | Change in axial length, spherical equivalent refractive error, adverse events, corneal erosion |
| Hiraoka 2018 [10] | Case-control study | Japan | Child | 182 | At least 10 years | Alpha ORTHO-K (ALPHA Corp., Nagoya, Japan) | Soft contact lenses (various; Manufacturers: Johnson & Johnson K.K.,Tokyo, Japan, or Bausch+ Lomb Japan, or Menicon, Japan, or CooperVision Japan) | Spherical equivalent refractive error, change in spherical equivalent refractive error, adverse events, corneal infiltrates, corneal erosion, |
| Jiang 2021 [11] | Prospective cohort study – OK group historical | China | Child | 71 | 1 year | Euclid Systems Ortho-K (Euclid Systems Corp., Herndon, VA, United States) | MFCL (BioThin, Bio Optic Inc., Taiwan, China) | Change in axial length |
| Li 2017, Li 2018 [12-13] | Prospective cohort study | China | Child | 50 | 13 months | Euclid Systems Ortho-K (Euclid SystemCorp., Herndon, USA) | Single-vision spectacles (brand not reported) | Change in axial length, apical corneal power |
| Na 2018 [14] | Case-control study | Korea | Child | 45 | 2 years | Ortho-K LK Lens (Lucid Korea) | No treatment | Axial length, change in axial length, spherical equivalent refractive error, corneal curvature (flatter meridian), corneal curvature (steeper meridian) |
| Nakamura 2021 [15] | Pooled data from three prospective cohort studies | Japan | Child | 194 | 2 years | Menicon Z Night Contact Lens (Menicon Co.)  αORTHO®-K lens (Alpha Corporation)  Emerald™ (Euclid Systems Corporation) | Single vision spectacles (brands not reported) | Change in axial length, change in spherical equivalent refractive error |
| Queirós 2010 [16] | Prospective cohort study | Spain | Adult | 28 | >3 years | Paragon CRT 100 (Paragon Vision Sciences, Mesa, AZ, USA) | LASIK | Central corneal power, change in corneal power |
| Queirós 2012 [17] | Prospective cohort study | Spain | Adult | 217 | At least 3 months | Not reported | Spectacles (brand not reported); LASIK; no treatment (non-myopic) | Satisfaction with correction, overall NEI RQL-42 score |
| Santodomingo Rubido 2009, 2012a,b, 2017 [18-21] | Prospective cohort study | Spain | Child | 61 | 2 years, +5 years of continued wear or discontinuation | Menicon Z Night (Menicon Co., Ltd, Nagoya, Japan) | Single-vision spectacles | Axial length, spherical refraction, refractive cylinder, flatter corneal meridian power, corneal curvature (steeper meridian), adverse events, corneal staining, dimple veiling, bacterial conjunctivitis |
| Tsai 2019 [22] | Case-control | Taiwan | Child | 61 | 2.01 +/- 1.48 years | Hiline (Macro Vision, Taipei) | Non-myopic | Adverse events |
| Turnbull 2016 [23] | Case-control study | New Zealand | Child | 110 | 1 year | Paragon CRT (Paragon CRT, Arizona, USA) or custom-made Falco (Tagerwilin, Switzerland) | Daily disposable MiSight (CooperVision, Pleasanton, USA) or custom-made contact lenses; or no treatment | Change in axial length, change in spherical refraction, best-corrected visual acuity, adverse events, lens binding |
| Walline 2009 [24] | Historically controlled trial | USA | Child | 40 | 2 years | Paragon CRT 100 (Paragon Vision Sciences, Mesa, AZ, USA) | Focus 2-week disposable contact lenses | Axial length |
| Yang 2021 [25] | Cohort study | China | Child | 40 | 12-18 months | Not reported | Single vision spectacles | Satisfaction with correction – overall PREP questionnaire score |
| Zhang 2019 [26] | Case-control study | China | Child | 98 in groups relevant to review | At least 2 years | LK lenses (Lucid, Bonghwa, Korea) or Euclid Systems Ortho-K (Euclid Systems, Herndon, VA) | Spectacles (type and brand not reported) | Axial length, change in axial length |
| Zhao 2021 [27] | Prospective Cohort Study | China | Child | 80 | 1 year | Not reported | spectacles (type and brand not reported) | Axial length, Change in axial length, spherical equivalent refractive error, Schirmer’s test |
| Zhu 2014 [28] | Case-control study | China | Child | 128 | At least 2 years | Euclid Systems Ortho-K (Euclid SystemCorp., Herndon, USA) | Single-vision spectacles | Axial length, change in axial length, spherical equivalent refractive error, corneal staining |

## **References**

1. Carracedo G, González-Méijome JM, Pintor J. Changes in diadenosine polyphosphates during alignment-fit and orthokeratology rigid gas permeable lens wear. *Investigative Ophthalmology and Visual Science*. 2012;53(8):4426-4432.

2. Chen Z, Niu L, Xue F, et al. Impact of pupil diameter on axial growth in orthokeratology. *Optometry and vision science : official publication of the American Academy of Optometry*. 2012;89(11):1636-1640.

3. Cheung SW, M.V. B, Cho P. Pre-treatment observation of axial elongation for evidence-based selection of children in Hong Kong for myopia control. *Contact Lens and Anterior Eye*. 2018;

4. Cho P, Cheung SW, Edwards M. The longitudinal orthokeratology research in children (LORIC) in Hong Kong: A pilot study on refractive changes and myopic control. *Current eye research*. 2005;30(1):71-80.

5. García-Porta N, Rico-del-Viejo L, Martin-Gil A, Carracedo G, Pintor J, González-Méijome JM. Differences in dry eye questionnaire symptoms in two different modalities of contact lens wear: Silicone-hydrogel in daily wear basis and overnight orthokeratology. *Biomed research international*. 2016;2016

6. Goldstone RN, Yildiz EH, Fan VC, Asbell PA. Changes in higher order wavefront aberrations after contact lens corneal refractive therapy and LASIK surgery. *Journal of Refractive Surgery*. 2009;25(5):1-8.

7. González-Pérez J, Sánchez-García Á, Villa-Collar C. Vision-specific quality of life: laser-assisted in situ keratomileusis versus overnight contact lens wear. *Eye & contact lens*. 2019;45(1):34-39.

8. He M, Du Y, Liu Q, et al. Effects of orthokeratology on the progression of low to moderate myopia in Chinese children. *BMC ophthalmology*. 2016;16:126-126.

9. Hiraoka T, Kakita T, Okamoto F, Takahashi H, Oshika T. Long-term effect of overnight orthokeratology on axial length elongation in childhood myopia: A 5-year follow-up study. *Investigative Ophthalmology and Visual Science*. 2012;53(7):3913-3919.

10. Hiraoka T, Sekine Y, Okamoto F, Mihashi T, Oshika T. Safety and efficacy following 10-years of overnight orthokeratology for myopia control. *Ophthalmic Physiol Opt*. 2018;38(3):281-289.

11. Jiang F, Huang X, Xia H, et al. The Spatial Distribution of Relative Corneal Refractive Power Shift and Axial Growth in Myopic Children: Orthokeratology Versus Multifocal Contact Lens. *Front Neurosci*. 2021;15:686932. doi:10.3389/fnins.2021.686932

12. Li Z, Cui D, Hu Y, Ao S, Zeng J, Yang X. Choroidal thickness and axial length changes in myopic children treated with orthokeratology. *Contact Lens and Anterior Eye*. 2017;40(6):417-423.

13. Li Z, Hu Y, Cui D, Long W, He M, Yang X. Change in subfoveal choroidal thickness secondary to orthokeratology and its cessation: a predictor for the change in axial length. *Acta ophthalmologica*. 2018;

14. Na M, Yoo A. The effect of orthokeratology on axial length elongation in children with myopia: Contralateral comparison study. *Japanese journal of ophthalmology*. 2018;62(3):327-334.

15. Nakamura Y, Hieda O, Yokota I, Teramukai S, Sotozono C, Kinoshita S. Comparison of myopia progression between children wearing three types of orthokeratology lenses and children wearing single-vision spectacles. *Jpn J Ophthalmol*. Sep 2021;65(5):632-643. doi:10.1007/s10384-021-00854-4

16. Queirós A, González-Méijome JM, Villa-Collar C, Gutierrez JR, Jorge J. Local steepening in peripheral corneal curvature after corneal refractive therapy and LASIK. *Optometry and vision science : official publication of the American Academy of Optometry*. 2010;87(6):432-439.

17. Queirós A, Villa-Collar C, Gutiérrez AR, Jorge J, González-Méijome JM. Quality of life of myopic subjects with different methods of visual correction using the NEI RQL-42 questionnaire. *Eye and Contact Lens*. 2012;38(2):116-121.

18. Santodomingo-Rubido J, Villa-Collar C, Gilmartin B, Gutiérrez-Ortega R. Myopia control with orthokeratology contact lenses in Spain (MCOS): study design and general baseline characteristics. *Journal of Optometry*. 2009;2(4):215-222.

19. Santodomingo-Rubido J, Villa-Collar C, Gilmartin B, Gutiérrez-Ortega R. Myopia control with orthokeratology contact lenses in Spain: refractive and biometric changes. *Investigative ophthalmology & visual science*. 2012;53(8):5060‐5065-5060‐5065.

20. Santodomingo-Rubido J, Villa-Collar C, Gilmartin B, Gutiérrez-Ortega R. Orthokeratology vs. spectacles: adverse events and discontinuations. *Optom Vis Sci*. 2012;89(8):1133-1139.

21. Santodomingo-Rubido J, Villa-Collar C, Gilmartin B, Gutiérrez-Ortega R, Sugimoto K. Long-term efficacy of orthokeratology contact lens wear in controlling the progression of childhood myopia. *Curr Eye Res*. 2017;42(5):713-720.

22. Tsai WS, Wang JH, Lee YC, Chiu CJ. Assessing the change of anisometropia in unilateral myopic children receiving monocular orthokeratology treatment. *Journal of the Formosan Medical Association*. 2019;

23. Turnbull PR, Munro OJ, Phillips JR. Contact lens methods for clinical myopia control. *Optometry and vision science : official publication of the American Academy of Optometry*. 2016;93(9):1120-1126.

24. Walline JJ, Jones LA, Sinnott LT. Corneal reshaping and myopia progression. *British Journal of Ophthalmology*. 2009;93(9):1181-1185.

25. Yang B, Ma X, Liu L, Cho P. Vision-related quality of life of Chinese children undergoing orthokeratology treatment compared to single vision spectacles. *Cont Lens Anterior Eye*. Aug 2021;44(4):101350. doi:10.1016/j.clae.2020.07.001

26. Zhang Y, Chen Y. Effect of orthokeratology on axial length elongation in anisomyopic children. *Optometry and vision science : official publication of the American Academy of Optometry*. 2019;96(1):43-47.

27. Zhao Q, Hao Q. Clinical efficacy of 0.01% atropine in retarding the progression of myopia in children. *Int Ophthalmol*. Mar 2021;41(3):1011-1017. doi:10.1007/s10792-020-01658-0

28. Zhu MJ, Feng HY, He XG, Zou HD, Zhu JF. The control effect of orthokeratology on axial length elongation in Chinese children with myopia. *BMC ophthalmology*. 2014;14:141-141.

## **Supplementary Table 3. Change in axial length in orthokeratology (OK) vs comparator treatments**. All studies contributing data contained paediatric participants only

| **Comparison** | **Time point** | **Studies (n)** | **Participants (n)** | **Effect Estimate** | |
| --- | --- | --- | --- | --- | --- |
| OK vs non-OK | 6 months | 10 [1-10] | 598 | Mean Difference (IV, Random, 95% CI [mm]) | -0.07 [-0.13, -0.02] |
|  | 1 year | 12 [1-3, 5, 8-15] | 972 | Mean Difference (IV, Random, 95% CI [mm]) | -0.16 [-0.25, -0.07] |
|  | 18 months | 4 [1-3,9] | 242 | Mean Difference (IV, Random, 95% CI [mm]) | -0.15 [-0.28, 0.02] |
|  | 2 years | 7 [1-3, 5, 12, 14, 16] | 521 | Mean Difference (IV, Random, 95% CI [mm]) | -0.19 [-0.29, -0.09] |
| OK vs spectacles | 6 months | 7 [1-4, 7, 9, 10] | 410 | Mean Difference (IV, Random, 95% CI [mm]) | -0.10 [-0.14, -0.06] |
|  | 1 year | 8 [1-3, 9-12, 14] | 642 | Mean Difference (IV, Random, 95% CI [mm]) | -0.18 [-0.25, -0.11] |
|  | 18 months | 4 [1-3, 9] | 242 | Mean Difference (IV, Random, 95% CI [mm]) | -0.15 [-0.28, -0.02] |
|  | 2 years | 6 [1-3, 12, 14, 16] | 463 | Mean Difference (IV, Random, 95% CI [mm]) | -0.16 [-0.28, -0.05] |

**References**

1. Chen Z, Niu L, Xue F, et al. Impact of pupil diameter on axial growth in orthokeratology. *Optometry and vision science : official publication of the American Academy of Optometry*. 2012;89(11):1636-1640.

2. Cho P, Cheung SW, Edwards M. The longitudinal orthokeratology research in children (LORIC) in Hong Kong: A pilot study on refractive changes and myopic control. *Current eye research*. 2005;30(1):71-80.

3. Cho P, Cheung SW. Retardation of myopia in Orthokeratology (ROMIO) study: a 2-year randomized clinical trial. *Invest Ophthalmol Vis Sci*. 2012;53(11):7077-7085.

4. Li Z, Cui D, Hu Y, Ao S, Zeng J, Yang X. Choroidal thickness and axial length changes in myopic children treated with orthokeratology. *Contact Lens and Anterior Eye*. 2017;40(6):417-423.

5. Na M, Yoo A. The effect of orthokeratology on axial length elongation in children with myopia: Contralateral comparison study. *Japanese journal of ophthalmology*. 2018;62(3):327-334.

6. Swarbrick HA, Alharbi A, Watt K, Lum E, Kang P. Myopia control during orthokeratology lens wear in children using a novel study design. *Ophthalmology*. 2015;122(3):620-630.

7. Cheung SW, Cho P. Validity of axial length measurements for monitoring myopic progression in orthokeratology. *Invest Ophthalmol Vis Sci*. 2013;54(3):1613-1615.

8. Hao Q, Zhao Q. Changes in subfoveal choroidal thickness in myopic children with 0.01% atropine, orthokeratology, or their combination. *Int Ophthalmol*. Sep 2021;41(9):2963-2971. doi:10.1007/s10792-021-01855-5

9. Jakobsen TM, Møller F. Control of myopia using orthokeratology lenses in Scandinavian children aged 6 to 12 years. Eighteen-month data from the Danish Randomized Study: Clinical study Of Near-sightedness; TReatment with Orthokeratology Lenses (CONTROL study). *Acta Ophthalmol*. Jul 7 2021;doi:10.1111/aos.14911

10. Zhao Q, Hao Q. Clinical efficacy of 0.01% atropine in retarding the progression of myopia in children. *Int Ophthalmol*. Mar 2021;41(3):1011-1017. doi:10.1007/s10792-020-01658-0

11. He M, Du Y, Liu Q, et al. Effects of orthokeratology on the progression of low to moderate myopia in Chinese children. *BMC ophthalmology*. 2016;16:126-126.

12. Hiraoka T, Kakita T, Okamoto F, Takahashi H, Oshika T. Long-term effect of overnight orthokeratology on axial length elongation in childhood myopia: A 5-year follow-up study. *Investigative Ophthalmology and Visual Science*. 2012;53(7):3913-3919.

13. Turnbull PR, Munro OJ, Phillips JR. Contact lens methods for clinical myopia control. *Optometry and vision science : official publication of the American Academy of Optometry*. 2016;93(9):1120-1126.

14. Zhu MJ, Feng HY, He XG, Zou HD, Zhu JF. The control effect of orthokeratology on axial length elongation in Chinese children with myopia. *BMC ophthalmology*. 2014;14:141-141.

15. Jiang F, Huang X, Xia H, et al. The Spatial Distribution of Relative Corneal Refractive Power Shift and Axial Growth in Myopic Children: Orthokeratology Versus Multifocal Contact Lens. *Front Neurosci*. 2021;15:686932. doi:10.3389/fnins.2021.686932

16. Nakamura Y, Hieda O, Yokota I, Teramukai S, Sotozono C, Kinoshita S. Comparison of myopia progression between children wearing three types of orthokeratology lenses and children wearing single-vision spectacles. *Jpn J Ophthalmol*. Sep 2021;65(5):632-643. doi:10.1007/s10384-021-00854-4

**Supplementary Table 4.** **Sensitivity analyses**. Meta-analyses between orthokeratology vs non-orthokeratology comparator treatments were repeated with data from either randomized studies (RCTs), non-randomized studies (non-RCTs) when sufficient data was available (data from n ≥ 2 studies). Meta-analysis also occurred if n ≥ 2 studies for the appropriate time point were available, criteria being a non-randomized study with a low or medium overall risk of bias, or a randomized study which did not systematically exclude non-responding participants. As all initial analyses used random effects models, sensitivity analyses also utilized a random effects model even when ≤ 2 studies were included, in order to show effects of excluding poorer quality studies on the initial model.

| **Time point** | **Comparison** | **Studies (n)** | **Participants (n)** | **Statistical Method** | **Effect Estimate** |
| --- | --- | --- | --- | --- | --- |
| 6 months | All studies | 10 [1-10] | 598 | Mean Difference (IV, Random, 95% CI [mm]) | -0.07 [-0.13, -0.02] |
|  | RCTs | 4 [3,6,8,9] | 225 | Mean Difference (IV, Random, 95% CI [mm]) | -0.05 [-0.13, 0.03] |
|  | Non-RCTs | 6 [1,2,4,5,10] | 373 | Mean Difference (IV, Random, 95% CI [mm]) | -0.09 [-0.15, -0.04] |
|  | High quality | 3 [2,6,9] | 171 | Mean Difference (IV, Random, 95% CI [mm]) | -0.11 [-0.22, 0.00] |
| 1 year | All studies | 12 [1-3,5,8-15] | 972 | Mean Difference (IV, Random, 95% CI [mm]) | -0.16 [-0.25, -0.07] |
|  | Non-RCTs | 9 [1,2,5,10-15] | 808 | Mean Difference (IV, Random, 95% CI [mm]) | -0.18 [-0.24, -0.11] |
|  | RCTs | 3 [2,8,9] | 164 | Mean Difference (IV, Random, 95% CI [mm]) | -0.09 [-0.30, 0.12] |
|  | High quality | 2 [2,9] | 118 | Mean Difference (IV, Random, 95% CI [mm]) | -0.18 [-0.24, -0.12] |
| 18 months | All studies | 4 [1-3,9] | 242 | Mean Difference (IV, Random, 95% CI [mm]) | -0.15 [-0.28, 0.02] |
|  | Non-RCTs | 2 [1-2] | 117 | Mean Difference (IV, Random, 95% CI [mm]) | -0.08 [-0.35, 0.19] |
| 2 years | All studies | 7 [1-3,5,12,14,16] | 521 | Mean Difference (IV, Random, 95% CI [mm]) | -0.19 [-0.29, -0.09] |
|  | Non-RCTs | 6 [1,2,5,12,14,16] | 451 | Mean Difference (IV, Random, 95% CI [mm]) | -0.18 [-0.29, -0.07] |

**References**

1. Chen Z, Niu L, Xue F, et al. Impact of pupil diameter on axial growth in orthokeratology. *Optometry and vision science : official publication of the American Academy of Optometry*. 2012;89(11):1636-1640.

2. Cho P, Cheung SW, Edwards M. The longitudinal orthokeratology research in children (LORIC) in Hong Kong: A pilot study on refractive changes and myopic control. *Current eye research*. 2005;30(1):71-80.

3. Cho P, Cheung SW. Retardation of myopia in Orthokeratology (ROMIO) study: a 2-year randomized clinical trial. *Invest Ophthalmol Vis Sci*. 2012;53(11):7077-7085.

4. Li Z, Cui D, Hu Y, Ao S, Zeng J, Yang X. Choroidal thickness and axial length changes in myopic children treated with orthokeratology. *Contact Lens and Anterior Eye*. 2017;40(6):417-423.

5. Na M, Yoo A. The effect of orthokeratology on axial length elongation in children with myopia: Contralateral comparison study. *Japanese journal of ophthalmology*. 2018;62(3):327-334.

6. Swarbrick HA, Alharbi A, Watt K, Lum E, Kang P. Myopia control during orthokeratology lens wear in children using a novel study design. *Ophthalmology*. 2015;122(3):620-630.

7. Cheung SW, Cho P. Validity of axial length measurements for monitoring myopic progression in orthokeratology. *Invest Ophthalmol Vis Sci*. 2013;54(3):1613-1615.

8. Hao Q, Zhao Q. Changes in subfoveal choroidal thickness in myopic children with 0.01% atropine, orthokeratology, or their combination. *Int Ophthalmol*. Sep 2021;41(9):2963-2971. doi:10.1007/s10792-021-01855-5

9. Jakobsen TM, Møller F. Control of myopia using orthokeratology lenses in Scandinavian children aged 6 to 12 years. Eighteen-month data from the Danish Randomized Study: Clinical study Of Near-sightedness; TReatment with Orthokeratology Lenses (CONTROL study). *Acta Ophthalmol*. Jul 7 2021;doi:10.1111/aos.14911

10. Zhao Q, Hao Q. Clinical efficacy of 0.01% atropine in retarding the progression of myopia in children. *Int Ophthalmol*. Mar 2021;41(3):1011-1017. doi:10.1007/s10792-020-01658-0

11. He M, Du Y, Liu Q, et al. Effects of orthokeratology on the progression of low to moderate myopia in Chinese children. *BMC ophthalmology*. 2016;16:126-126.

12. Hiraoka T, Kakita T, Okamoto F, Takahashi H, Oshika T. Long-term effect of overnight orthokeratology on axial length elongation in childhood myopia: A 5-year follow-up study. *Investigative Ophthalmology and Visual Science*. 2012;53(7):3913-3919.

13. Turnbull PR, Munro OJ, Phillips JR. Contact lens methods for clinical myopia control. *Optometry and vision science : official publication of the American Academy of Optometry*. 2016;93(9):1120-1126.

14. Zhu MJ, Feng HY, He XG, Zou HD, Zhu JF. The control effect of orthokeratology on axial length elongation in Chinese children with myopia. *BMC ophthalmology*. 2014;14:141-141.

15. Jiang F, Huang X, Xia H, et al. The Spatial Distribution of Relative Corneal Refractive Power Shift and Axial Growth in Myopic Children: Orthokeratology Versus Multifocal Contact Lens. *Front Neurosci*. 2021;15:686932. doi:10.3389/fnins.2021.686932

16. Nakamura Y, Hieda O, Yokota I, Teramukai S, Sotozono C, Kinoshita S. Comparison of myopia progression between children wearing three types of orthokeratology lenses and children wearing single-vision spectacles. *Jpn J Ophthalmol*. Sep 2021;65(5):632-643. doi:10.1007/s10384-021-00854-4

**Supplementary Table 5. Axial length in orthokeratology (OK) vs comparator treatments**. All studies contributing data contained pediatric participants only.

| **Comparison** | **Time point** | **Studies (n)** | **Participants (n)** | **Statistical Method** | **Effect Estimate** |
| --- | --- | --- | --- | --- | --- |
| OK vs non-OK | 6 months | 2 [1,2] | 97 | Mean Difference (IV, Fixed, 95% CI [mm]) | 0.08 [-0.24, 0.39] |
|  | 1 year | 8 [1-8] | 767 | Mean Difference (IV, Random, 95% CI [mm]) | -0.03 [-0.19, 0.13] |
|  | 2 years | 5 [1,4-7] | 311 | Mean Difference (IV, Fixed, 95% CI [mm]) | -0.13 [-0.32, 0.05] |
| OK vs spectacles | 6 months | 2 [1,2] | 97 | Mean Difference (IV, Fixed, 95% CI [mm]) | 0.08 [-0.24, 0.39] |
|  | 1 year | 5 [1-3,6,7] | 510 | Mean Difference (IV, Fixed, 95% CI [mm]) | -0.19 [-0.32, -0.06] |
|  | 2 years | 3 [1,6,7] | 197 | Mean Difference (IV, Fixed, 95% CI [mm]) | -0.23 [-0.44, -0.01] |

**References**

1. Santodomingo-Rubido J, Villa-Collar C, Gilmartin B, Gutiérrez-Ortega R. Myopia control with orthokeratology contact lenses in Spain: refractive and biometric changes. *Investigative ophthalmology & visual science*. 2012;53(8):5060‐5065-5060‐5065.

2. Zhao Q, Hao Q. Clinical efficacy of 0.01% atropine in retarding the progression of myopia in children. *Int Ophthalmol*. Mar 2021;41(3):1011-1017. doi:10.1007/s10792-020-01658-0

3. He M, Du Y, Liu Q, et al. Effects of orthokeratology on the progression of low to moderate myopia in Chinese children. *BMC ophthalmology*. 2016;16:126-126.

4. Na M, Yoo A. The effect of orthokeratology on axial length elongation in children with myopia: Contralateral comparison study. *Japanese journal of ophthalmology*. 2018;62(3):327-334.

5. Walline JJ, Jones LA, Sinnott LT. Corneal reshaping and myopia progression. *British Journal of Ophthalmology*. 2009;93(9):1181-1185.

6. Zhu MJ, Feng HY, He XG, Zou HD, Zhu JF. The control effect of orthokeratology on axial length elongation in Chinese children with myopia. *BMC ophthalmology*. 2014;14:141-141.

7. Zhang Y, Chen Y. Effect of orthokeratology on axial length elongation in anisomyopic children. *Optometry and vision science : official publication of the American Academy of Optometry*. 2019;96(1):43-47.

8. Jiang F, Huang X, Xia H, et al. The Spatial Distribution of Relative Corneal Refractive Power Shift and Axial Growth in Myopic Children: Orthokeratology Versus Multifocal Contact Lens. *Front Neurosci*. 2021;15:686932. doi:10.3389/fnins.2021.686932

**Supplementary Table 6.** **Change in axial length following discontinuation of orthokeratology (OK) vs ongoing OK treatment, or treatment prior to discontinuation**. Time point refers to number of months of discontinuation of OK treatment. mm = millimetres.

| **Comparison** | **Time point** | **Studies (n)** | **Participants (n)** | **Statistical Method** | **Effect Estimate** |
| --- | --- | --- | --- | --- | --- |
| Discontinuation vs OK (parallel group) | 6-7 months | 2 [1,2] | 79 | Mean Difference (IV, Fixed, 95% CI [mm]) | 0.10 [0.06, 0.14] |
| Discontinuation vs OK (crossover) | 6-7 months | 2 [1,2] | 81 | Mean Difference (IV, Fixed, 95% CI [mm]) | 0.10 [0.06, 0.13] |
| Discontinuation vs non-OK | 6-7 months | 2 [1,2] | 78 | Mean Difference (IV, Fixed, 95% CI [mm]) | 0.06 [0.02, 0.10] |

**References**

1. Cho P, Cheung SW. Discontinuation of orthokeratology on eyeball elongation (DOEE). *Cont Lens Anterior Eye*. 2017;40(2):82-87.

2. Swarbrick HA, Alharbi A, Watt K, Lum E, Kang P. Myopia control during orthokeratology lens wear in children using a novel study design. *Ophthalmology*. 2015;122(3):620-630.

**Supplementary Table 7. Spherical equivalent refractive error in orthokeratology (OK) vs comparator treatments**.

| **Comparison** | **Time point** | **Studies (n)** | **Participants (n)** | **Age-group** | **Statistical Method** | **Effect Estimate** |
| --- | --- | --- | --- | --- | --- | --- |
| OK vs non-OK | 6 months | 3 [1-3] | 164 | Mixed | Mean Difference (IV, Random, 95% CI [D]) | 0.98 [-0.22, 2.18] |
|  | 6 months | 2 [2-4] | 182 | Paediatric | Mean Difference (IV, Random, 95% CI [D]) | 0.95 [-0.29, 2.18] |
|  | 1 year | 2 [2,4,5] | 214 | Paediatric | Mean Difference (IV, Random, 95% CI [D]) | 1.75 [0.38, 3.12] |
|  | 2 years | 2 [2,5] | 102 | Paediatric | Mean Difference (IV, Random, 95% CI [D]) | 2.47 [0.46, 4.49] |

**References**

1. Goldstone, R.N., et al., *Changes in higher order wavefront aberrations after contact lens corneal refractive therapy and LASIK surgery.* Journal of Refractive Surgery, 2009. **25**(5): p. 1-8.

2. Na, M. and A. Yoo, *The effect of orthokeratology on axial length elongation in children with myopia: Contralateral comparison study.* Japanese journal of ophthalmology, 2018. **62**(3): p. 327-334.

3. Swarbrick, H.A., et al., *Myopia control during orthokeratology lens wear in children using a novel study design.* Ophthalmology, 2015. **122**(3): p. 620-630.

4. Zhao, Q. and Q. Hao, *Clinical efficacy of 0.01% atropine in retarding the progression of myopia in children.* Int Ophthalmol, 2021. **41**(3): p. 1011-1017.

5. Zhu, M.J., et al., *The control effect of orthokeratology on axial length elongation in Chinese children with myopia.* BMC ophthalmology, 2014. **14**: p. 141-141.

**Supplementary Table 8. Corneal measurements in orthokeratology (OK) vs comparator treatments**. All studies contributing data contained pediatric participants only.

D = diopters, mm = millimeters.

| **Comparison** | **Time point** | **Studies (n)** | **Participants (n)** | **Statistical Method** | **Effect Estimate** |
| --- | --- | --- | --- | --- | --- |
| **Corneal curvature**  **(Flatter meridian)** |  |  |  |  |  |
| OK vs non-OK | 6 months | 2 [1,2] | 109 | Mean Difference (IV, Fixed, 95% CI [D]) | -1.83 [-2.32, -1.34] |
| **Corneal curvature**  **(Steeper meridian)** |  |  |  |  |  |
| OK vs non-OK | 6 months | 2 [1,2] | 109 | Mean Difference (IV, Fixed, 95% CI [D]) | -1.85 [-2.39, -1.32] |
| **Change in central corneal thickness** |  |  |  |  |  |
| OK vs spectacles | 6 months | 2 [3,4] | 126 | Mean Difference (IV, Fixed, 95% CI [mm]) | -0.01 [-0.01, -0.01] |

**References**

1. Swarbrick HA, Alharbi A, Watt K, Lum E, Kang P. Myopia control during orthokeratology lens wear in children using a novel study design. *Ophthalmology*. 2015;122(3):620-630.

2. Santodomingo-Rubido J, Villa-Collar C, Gilmartin B, Gutiérrez-Ortega R. Myopia control with orthokeratology contact lenses in Spain: refractive and biometric changes. *Investigative ophthalmology & visual science*. 2012;53(8):5060‐5065-5060‐5065.

3. Li Z, Cui D, Hu Y, Ao S, Zeng J, Yang X. Choroidal thickness and axial length changes in myopic children treated with orthokeratology. *Contact Lens and Anterior Eye*. 2017;40(6):417-423.

4. Cheung SW, Cho P. Validity of axial length measurements for monitoring myopic progression in orthokeratology. *Invest Ophthalmol Vis Sci*. 2013;54(3):1613-1615.

**Supplementary Table 9. Patient satisfaction in orthokeratology (OK) vs comparator treatments**. Patient satisfaction score for all studies was rated using the National Eye Institute Refractive Error Quality of Life instrument (NEI-RQL). SCL = soft contact lenses.

| **Comparison** | **Time point** | **Studies (n)** | **Participants (n)** | **Age-group** | **Statistical Method** | **Effect Estimate** |
| --- | --- | --- | --- | --- | --- | --- |
| OK vs SCL | 3 months | 2 [1,2] | 99 | Adult | Mean Difference (IV, Fixed, 95% CI) | 4.97 [-1.68, 11.62] |

**References**

1. Queirós A, Villa-Collar C, Gutiérrez AR, Jorge J, González-Méijome JM. Quality of life of myopic subjects with different methods of visual correction using the NEI RQL-42 questionnaire. *Eye and Contact Lens*. 2012;38(2):116-121.

2. Ritchey ER, Barr JT, Mitchell GL. The comparison of overnight lens modalities (COLM) Study. *Eye and Contact Lens*. 2005;31(2):70-75.

**Supplementary Table 10. Number of participants experiencing adverse events.** OK = Orthokeratology, CL = contact lenses, OR = odds ratio. All studies with pediatric participants utilized single vision spectacles or no treatment within comparator groups, whereas in studies with adult comparator groups wore CL.

| **Comparison** | **Time point** | **Studies (n)** | **Age group** | **OK** | | **Comparator** | | **Method** | **Effect Estimate** |
| --- | --- | --- | --- | --- | --- | --- | --- | --- | --- |
|  |  |  |  | **Events** | **Participants** | **Events** | **Participants** |  |  |
| OK vs non-OK | Overall | 7 [1-7] | Paediatric and adult | 57 | 239 | 19 | 239 | OR IV, Fixed, 95% CI | 7.36 [3.31, 16.37] |
|  | 12 months | 2 [2,5] | Adult | 32 | 83 | 18 | 84 | OR IV, Fixed, 95% CI | 5.2 [1.77, 15.33] |
| OK vs other CL | 12 months | 2 [2,5] | Adult | 32 | 83 | 18 | 63 | OR IV, Fixed, 95% CI | 4.59 [1.53, 13.76] |
| OK vs non-CL | Overall | 4 [1,3,4,6] | Paediatric | 23 | 135 | 1 | 127 | OR IV, Fixed, 95% CI | 10.74 [2.86, 40.27] |
| OK vs spectacles | Overall | 4 [1,3,6,7] | Paediatric | 20 | 125 | 1 | 124 | OR IV, Fixed, 95% CI | 9.65 [2.54, 36.64] |
|  | 24 months | 2 [3,6] | Paediatric | 14 | 82 | 1 | 85 | OR IV, Fixed, 95% CI | 10.08 [1.83, 55.41] |

**References**

1. Hiraoka T, Kakita T, Okamoto F, Takahashi H, Oshika T. Long-term effect of overnight orthokeratology on axial length elongation in childhood myopia: A 5-year follow-up study. *Investigative Ophthalmology and Visual Science*. 2012;53(7):3913-3919.

2. Polse KA, Brand RJ, Keener RJ, Schwalbe JS, Vastine DW. The Berkeley Orthokeratology Study, part III: safety. *Am J Optom Physiol Opt*. 1983;60(4):321-328.

3. Santodomingo-Rubido J, Villa-Collar C, Gilmartin B, Gutiérrez-Ortega R. Orthokeratology vs. spectacles: adverse events and discontinuations. *Optom Vis Sci*. 2012;89(8):1133-1139.

4. Tsai WS, Wang JH, Lee YC, Chiu CJ. Assessing the change of anisometropia in unilateral myopic children receiving monocular orthokeratology treatment. *Journal of the Formosan Medical Association*. 2019;

5. Turnbull PR, Munro OJ, Phillips JR. Contact lens methods for clinical myopia control. *Optometry and vision science : official publication of the American Academy of Optometry*. 2016;93(9):1120-1126.

6. Cho P, Cheung SW. Retardation of myopia in Orthokeratology (ROMIO) study: a 2-year randomized clinical trial. *Invest Ophthalmol Vis Sci*. 2012;53(11):7077-7085.

7. Jakobsen TM, Møller F. Control of myopia using orthokeratology lenses in Scandinavian children aged 6 to 12 years. Eighteen-month data from the Danish Randomized Study: Clinical study Of Near-sightedness; TReatment with Orthokeratology Lenses (CONTROL study). *Acta Ophthalmol*. Jul 7 2021;doi:10.1111/aos.14911

**Supplementary Table 11. Incidence of adverse events between treatment groups.** OK = Orthokeratology, OR = odds ratio. Methods used to classify and rate degree of corneal staining included number of participants experiencing the event, staining grade and Cornea and Contact Lens Research Unit grading scales (CCLRU) grade.

| **Comparison** | **Time point** | **Studies (n)** | **Age group** | **OK** | | **Comparator** | | **Statistical Method** | **Effect Estimate** |
| --- | --- | --- | --- | --- | --- | --- | --- | --- | --- |
| **Incidence of any adverse event**  **(n eyes)** |  |  |  | **Events** | **Participants or eyes** | **Events** | **Participants or eyes** |  |  |
| OK vs non-OK | Overall | 2 [1-2] | Paediatric | 69 | 166 | 43 | 134 | OR IV, Random, 95% CI | 4.72 [0.11, 199.75] |
| **Incidence of Corneal erosion**  **(n participants)** |  |  |  |  |  |  |  |  |  |
| OK vs non-OK | Overall | 2 [1-3] | Paediatric | 2 | 126 | 1 | 99 | OR IV, Fixed, 95% CI | 1.42 [0.19, 10.78] |
| **Incidence of Corneal Staining**  **(n participants)** |  |  |  |  |  |  |  |  |  |
| OK vs spectacles | 2 years | 4 [4-7] | Paediatric | 30 | 187 | 0 | 173 | OR IV, Fixed, 95% CI | 14.98 [3.55, 63.10] |
| **CCLRU grade (corneal staining)** |  |  |  |  |  |  |  |  |  |
| OK vs non-OK | 1 month | 2 [8,9] | Adult | NA | 45 | NA | 53 | MD IV, Fixed, 95% CI | 0.37 [0.14, 0.61] |
| **Lens Binding**  **(n participants)** |  |  |  |  |  |  |  |  |  |
| OK vs non-OK | Overall | 2 [10,11] | Paediatric | 0 | 78 | 1 | 58 | OR IV, Fixed, 95% CI | 0.32 [0.01, 8.24] |

**References**

1. Hiraoka T, Sekine Y, Okamoto F, Mihashi T, Oshika T. Safety and efficacy following 10-years of overnight orthokeratology for myopia control. *Ophthalmic Physiol Opt*. 2018;38(3):281-289.

2. Santodomingo-Rubido J, Villa-Collar C, Gilmartin B, Gutiérrez-Ortega R. Orthokeratology vs. spectacles: adverse events and discontinuations. *Optom Vis Sci*. 2012;89(8):1133-1139.

3. Hiraoka T, Kakita T, Okamoto F, Takahashi H, Oshika T. Long-term effect of overnight orthokeratology on axial length elongation in childhood myopia: A 5-year follow-up study. *Investigative Ophthalmology and Visual Science*. 2012;53(7):3913-3919.

4. Jakobsen TM, Møller F. Control of myopia using orthokeratology lenses in Scandinavian children aged 6 to 12 years. Eighteen-month data from the Danish Randomized Study: Clinical study Of Near-sightedness; TReatment with Orthokeratology Lenses (CONTROL study). *Acta Ophthalmol*. Jul 7 2021;doi:10.1111/aos.14911

5. Cho P, Cheung SW. Retardation of myopia in Orthokeratology (ROMIO) study: a 2-year randomized clinical trial. *Invest Ophthalmol Vis Sci*. 2012;53(11):7077-7085.

6. Cho P, Cheung SW, Edwards M. The longitudinal orthokeratology research in children (LORIC) in Hong Kong: A pilot study on refractive changes and myopic control. *Current eye research*. 2005;30(1):71-80.

7. Zhu MJ, Feng HY, He XG, Zou HD, Zhu JF. The control effect of orthokeratology on axial length elongation in Chinese children with myopia. *BMC ophthalmology*. 2014;14:141-141.

8. Carracedo G, González-Méijome JM, Pintor J. Changes in diadenosine polyphosphates during alignment-fit and orthokeratology rigid gas permeable lens wear. *Investigative Ophthalmology and Visual Science*. 2012;53(8):4426-4432.

9. García-Porta N, Rico-del-Viejo L, Martin-Gil A, Carracedo G, Pintor J, González-Méijome JM. Differences in dry eye questionnaire symptoms in two different modalities of contact lens wear: Silicone-hydrogel in daily wear basis and overnight orthokeratology. *Biomed research international*. 2016;2016

10. Swarbrick HA, Alharbi A, Watt K, Lum E, Kang P. Myopia control during orthokeratology lens wear in children using a novel study design. *Ophthalmology*. 2015;122(3):620-630.

11. Turnbull PR, Munro OJ, Phillips JR. Contact lens methods for clinical myopia control. *Optometry and vision science : official publication of the American Academy of Optometry*. 2016;93(9):1120-1126.

**Supplementary Table 12. Incidence and severity of dry eye symptoms in orthokeratology (OK) vs comparator treatments.** OR = odds ratio, MD = mean difference. Dry eye was measured in tear volume (Shirmer test, in millimeters) and Dry Eye Questionnaire (DEQ) discomfort and dryness scores.

| **Comparison** | **Time Point** | **Studies (n)** | **Age group** | **Participants (n)** | **Statistical Method** | **Effect Estimate** |
| --- | --- | --- | --- | --- | --- | --- |
| **Tear volume** |  |  |  |  |  |  |
| OK vs non-OK | 1 month | 2 [1,2] | Adult | 98 | MD IV, Fixed, 95% CI | 4.32 [-0.06, 8.70] |
| **DEQ dryness score** |  |  |  |  |  |  |
| OK vs non-OK | 1 month | 2 [1,2] | Adult | 98 | MD IV, Fixed, 95% CI | 0.26, [-0.62, 0.10] |
| **DEQ discomfort score** |  |  |  |  |  |  |
| OK vs non-OK | 1 month | 2 [1,2] | Adult | 98 | MD IV, Fixed, 95% CI | 0.06 [-0.28, 0.39] |

**References**

1. Carracedo G, González-Méijome JM, Pintor J. Changes in diadenosine polyphosphates during alignment-fit and orthokeratology rigid gas permeable lens wear. *Investigative Ophthalmology and Visual Science*. 2012;53(8):4426-4432.

2. García-Porta N, Rico-del-Viejo L, Martin-Gil A, Carracedo G, Pintor J, González-Méijome JM. Differences in dry eye questionnaire symptoms in two different modalities of contact lens wear: Silicone-hydrogel in daily wear basis and overnight orthokeratology. *Biomed research international*. 2016;2016
